# Hsa_circ_0007321 regulates Zika virus replication through miR-492/NFKBID/NF-κB signaling pathway

**DOI:** 10.1128/jvi.01232-23

**Published:** 2023-12-05

**Authors:** Lan Kang, He Xie, Haiyan Ye, Andre J. Jeyarajan, Charlotte A. Warner, Yike Huang, Yaoqiang Shi, Yujia Li, Chunhui Yang, Min Xu, Wenyu Lin, Jujun Sun, Limin Chen, Xiaoqiong Duan, Shilin Li

**Affiliations:** 1 Institute of Blood Transfusion, Chinese Academy of Medical Sciences and Peking Union Medical College, Chengdu, Sichuan, China; 2 Chengdu Fifth People’s Hospital, Chengdu, Sichuan, China; 3 The Hospital of Xidian Group, Xian, Shaanxi, China; 4 Department of Laboratory Medicine, Chengdu Second People’s Hospital, Chengdu, Sichuan, China; 5 Liver Center and Gastrointestinal Division, Department of Medicine, Massachusetts General Hospital, Harvard Medical School, Boston, Massachusetts, USA; 6 Joint Laboratory on Transfusion-transmitted Infectious Diseases between Institute of Blood Transfusion, Chinese Academy of Medical Sciences and Nanning Blood Center, Nanning Blood Center, Key Laboratory for Transfusion-transmitted Infectious Diseases of the Health Commission of Nanning City, Nanning, Guangxi, China; University of North Carolina at Chapel Hill, Chapel Hill, North Carolina, USA

**Keywords:** hsa_circ_0007321, Zika virus, miR-492, NFKBID, NF-κB pathway

## Abstract

Circular RNAs (CircRNAs) have been shown to play a critical role in regulating viral infection and replication. We performed RNA sequencing to identify differentially expressed circRNAs in A549 human lung adenocarcinoma cells with and without Zika virus (ZIKV) infection. Notably, hsa_circ_0007321 was significantly downregulated after ZIKV infection. We then explored the role and mechanism of hsa_circ_0007321 in the regulation of ZIKV replication. Hsa_circ_0007321, derived from exons 2, 3, 4, and 5 of the DIS3L2 (DIS3 like 3'−5' exoribonuclease 2) gene, was mainly enriched in cytoplasm. We found that the knockdown of hsa_circ_0007321 significantly increased microRNA-492 levels to decrease NFKBID (NFKB inhibitor delta) expression, leading to the activation of the nuclear factor-κB (NF-κB) signaling pathway, inflammatory cytokine production, and subsequently, inhibition of ZIKV replication. These findings illustrate that hsa_circ_0007321 acts as a competitive endogenous RNA that regulates ZIKV replication through the miR-492/NFKBID NF-κB signaling pathway.

**IMPORTANCE:**

Over the past decade, increasing evidence has shown that circular RNAs (circRNAs) play important regulatory roles in viral infection and host antiviral responses. However, reports on the role of circRNAs in Zika virus (ZIKV) infection are limited. In this study, we identified 45 differentially expressed circRNAs in ZIKV-infected A549 cells by RNA sequencing. We clarified that a downregulated circRNA, hsa_circ_0007321, regulates ZIKV replication through targeting of miR-492 and the downstream gene NFKBID. NFKBID is a negative regulator of nuclear factor-κB (NF-κB), and we found that inhibition of the NF-κB pathway promotes ZIKV replication. Therefore, this finding that hsa_circ_0007321 exerts its regulatory role on ZIKV replication through the miR-492/NFKBID/NF-κB signaling pathway has implications for the development of strategies to suppress ZIKV and possibly other viral infections.

## INTRODUCTION

Zika virus (ZIKV) is a single-stranded positive-sense RNA virus belonging to the *Flavivirus* genus that is mainly transmitted by Aedes mosquitoes (1). The most recent ZIKV epidemic began in Brazil in March 2015 and then spread rapidly across South America and the rest of the Americas (2). This epidemic was of high concern because ZIKV infection in pregnant women could lead to congenital Zika syndrome, characterized by microcephaly in newborns. ZIKV infection can also lead to severe symptoms such as Guillain-Barre syndrome in adults (3), although in most cases, the infection is asymptomatic and self-limited. Recent studies have reported that ZIKV infection activated nuclear factor-κB (NF-κB)-mediated inflammatory reactions in Drosophila brain, which in turn limited viral proliferation (4). NF-κB is an important transcription factor that regulates several physiological processes including host antiviral immunity and proinflammatory responses. Some viruses can suppress NF-κB activation to escape from the immune response (5). The mammalian NF-κB signaling system consists of five proteins (RelA, c-Rel, RelB, p105/p50, and p100/p52). In resting cells, inactive NF-κB is sequestered in the cytoplasm by a family of IκB inhibitory proteins. Various stimuli such as viral infection induce phosphorylation- and ubiquitination-mediated degradation of IκB proteins, leading to the activation and nuclear translocation of NF-κB (5). NFKBID (NF-κB inhibitor delta, IκBδ) belongs to the IκB protein family and acts as an atypical NF-κB inhibitor by interacting with p50 (6–8). However, the interaction between ZIKV infection and the NF-κB pathway in human cells is still poorly understood.

Circular RNAs (CircRNAs) are a class of non-coding RNAs with a special covalent closed-loop structure, formed by back splicing of the pre-mRNA. CircRNAs have been reported to be involved in viral replication, antiviral immune responses, and the pathogenesis of infectious diseases (9–11). Many studies have shown that circRNAs can work as microRNA (miRNA) “sponges” to regulate target gene expression (12, 13). For example, circBCL2L1 functions as a competitive endogenous RNA (ceRNA) of TRAF6 (TNF receptor associated factor 6) by competitively binding to miR-30c-3-3p, thereby activating the NF-κB/IFN regulatory factor 3 (IRF3) inflammatory pathway and enhancing the innate immune response (14). Here, we performed RNA sequencing (RNA-seq) to analyze expression of circRNAs in A549 cells with and without ZIKV infection (accession number: GSE146423) and found that hsa_circ_0007321 was significantly downregulated after ZIKV infection. We further confirmed the downregulation of hsa_circ_0007321 by ZIKV infection in A549, as well as in the U251 human glioblastoma cells. Notably, hsa_circ_0007321 was mainly localized in the cytoplasm, and knockdown of hsa_circ_0007321 inhibited ZIKV replication through the miR-492/NFKBID axis and activation of the NF-κB signaling pathway. These findings demonstrate that hsa_circ_0007321 may serve as a potential target for the treatment of ZIKV infection.

## RESULTS

### Hsa_circ_0007321 was downregulated in ZIKV-infected cells

**Fig 1:**
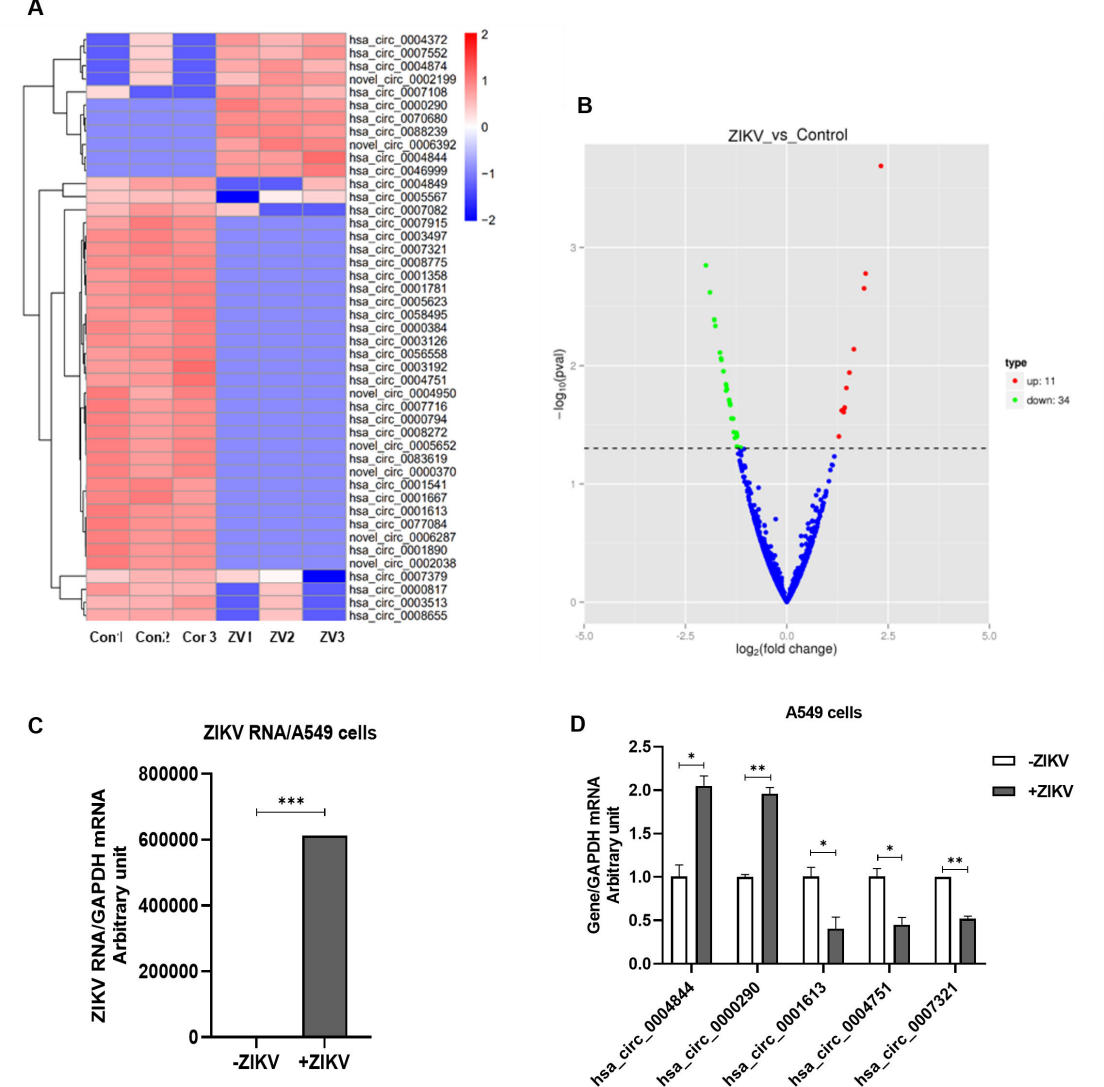
Identification and confirmation of differentially expressed circRNAs during ZIKV infection in A549 cells. A549 cells were infected with ZIKV by multiplicity of infection of 0.5. Total RNAs were extracted at 48 h post-infection for circRNA profiling by RNA-seq, and the data were compared with those obtained from uninfected A549 cells. Two upregulated and three downregulated circRNAs were selected and validated by qRT-PCR. *P < 0.05; < 0.01;***P<0.001. (**A**) Heat map of the circRNA expression in A549 and ZIKV-infected A549 cells. Red indicates upregulation, and blue indicates downregulation (fold change >2, *P* < 0.05). (**B**) Volcanic map of differentially expressed circRNAs between A549 and ZIKV-infected A549 cells. (**C**) ZIKV RNA was significantly increased in ZIKV-infected A549 cells compared to uninfected cells. (**D**) Verification of two upregulated and three downregulated circRNA expression in ZIKV-infected A549 cells. Hsa_circ_0004844 and hsa_circ_0000290 were significantly upregulated, while hsa_circ_0001613, hsa_circ_0004751, and hsa_circ_0007321 were significantly downregulated in ZIKV-infected A549 cells compared to uninfected control.

We performed RNA-seq in uninfected A549 cells and ZIKV-infected A549 cells at 48 h post-infection and profiled circRNA expression. We obtained 806 circRNAs; of which, 45 circRNAs were differentially expressed after ZIKV infection (fold change >2, *P* < 0.05), including 11 upregulated and 34 downregulated circRNAs (Fig. 1A and B). We selected two upregulated and three downregulated circRNAs and validated them by qRT-PCR. Consistent with the RNA-seq results, we found that ZIKV infection significantly increased expression of hsa_circ_0004844 and hsa_circ_0000290 to 2.05 ± 0.11-fold and 1.96 ± 0.09-fold, respectively, while hsa_circ_0001613, hsa_circ_0004751, and hsa_circ_0007321 were downregulated (Fig. 1D). Notably, hsa_circ_0007321 was markedly downregulated by 47.9% ± 2.2% in ZIKV-infected A549 cells.

**Fig 2:**
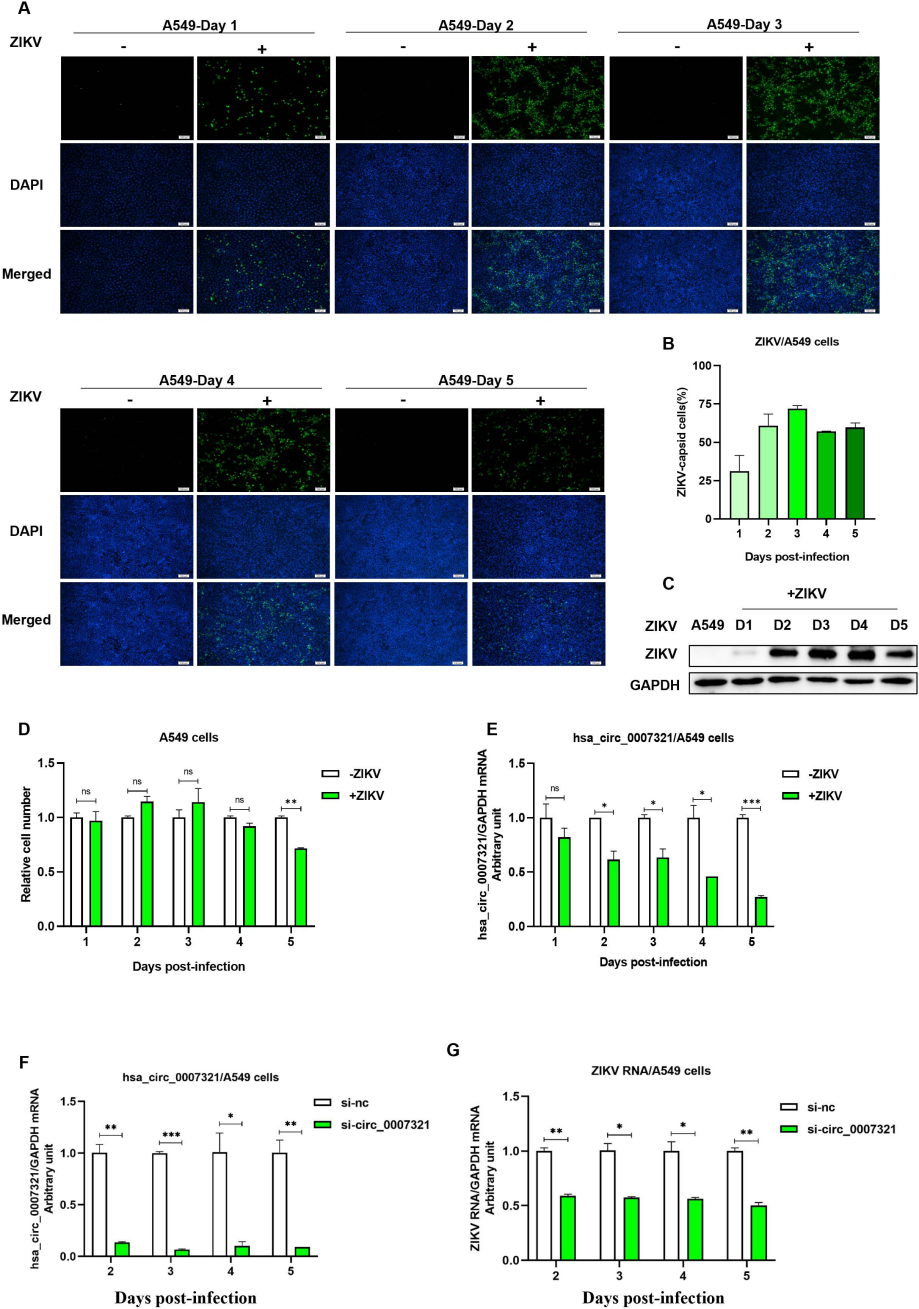
ZIKV infection downregulated hsa_circ_0007321 expression, which restricted ZIKV replication in A549 cells. (**A and B**) A549 cells were seeded with or without ZIKV infection by multiplicity of infection (MOI) of 0.5. At times ranging from 1 to 5 days, the cells were fixed and stained with fluorescent probes for DNA (DAPI; blue) and ZIKV capsid (green). Total cell numbers and ZIKV capsid-positive cells were counted, and the proportion of ZIKV capsid-positive cells was calculated. (**A**) Immunofluorescent staining of ZIKV capsid protein (Green) at 1–5 dpi. (**B**) Proportion of ZIKV capsid-positive cells in total cells of Fig. 2A. (**C–E**) A549 cells were infected with or without ZIKV by MOI of 0.5. Cells were collected to count cell numbers at day 1 to 5 dpi. Total RNAs and total proteins were extracted. The expression of ZIKV NS1 and hsa_circ_0007321 was detected by Western blot and qRT-PCR. (**C**)ZIKV NS1 significantly increased at 1–5 dpi. (**D**) Relative cell number of uninfected and ZIKV-infected A549 cells at 1–5 dpi. (**E**) Hsa_circ_0007321 was significantly downregulated in ZIKV-infected A549 cells compared to uninfected A549 cells at 1–5 dpi. (**F and G**) A549 cells were transfected with hsa_circ_0007321 siRNA or its corresponding negative control (si-nc) for 24 h, followed by ZIKV infection at an MOI of 0.5. ZIKV RNA and hsa_circ_0007321 expression were detected using qRT-PCR at 2–5 dpi. (**F**) si-circ_0007321 significantly knocked down hsa_circ_0007321 in ZIKV-infected A549 cells at 2–5 dpi. (**G**) Knockdown of hsa_circ_0007321 significantly inhibited ZIKV RNA replication at 2–5 dpi. ns represents *P* > 0.05; *P < 0.05; **P < 0.01; ***P < 0.001.

We next investigated the association between hsa_circ_0007321 expression and ZIKV infection and replication at 1–5 days post-infection (dpi). ZIKV infection was confirmed by ZIKV capsid and NS1 protein levels, which were significantly elevated after infection (Fig. 2A through C). We found that the ratio of ZIKV-infected/total A549 cells increased from 31% ± 10.3% at 1 dpi to 71.8% ± 2.12% at 3 dpi and stabilized at 57% ± 3.0% at 4–5 dpi (Fig. 2A and B). Meanwhile, there was not a significant difference in total cell numbers between ZIKV-infected and uninfected A549 cells at 1–4 dpi. There was, however, a significant difference in cell numbers between ZIKV-infected cells and A549 cells at 5 dpi (Fig. 2D). We, therefore, normalized the relative expression of hsa_circ_0007321 levels using GAPDH as an internal background control to obtain an arbitrary unit. We found that relative hsa_circ_0007321 arbitrary units significantly decreased in ZIKV-infected cells compared to the uninfected A549 cells at 1–5 dpi, in a time-dependent manner (Fig. 2E). Moreover, we found that knockdown of hsa_circ_0007321 significantly reduced ZIKV RNA replication at 2–5 dpi (Fig. 2F through G).

### Characteristics of hsa_circ_0007321

**Fig 3:**
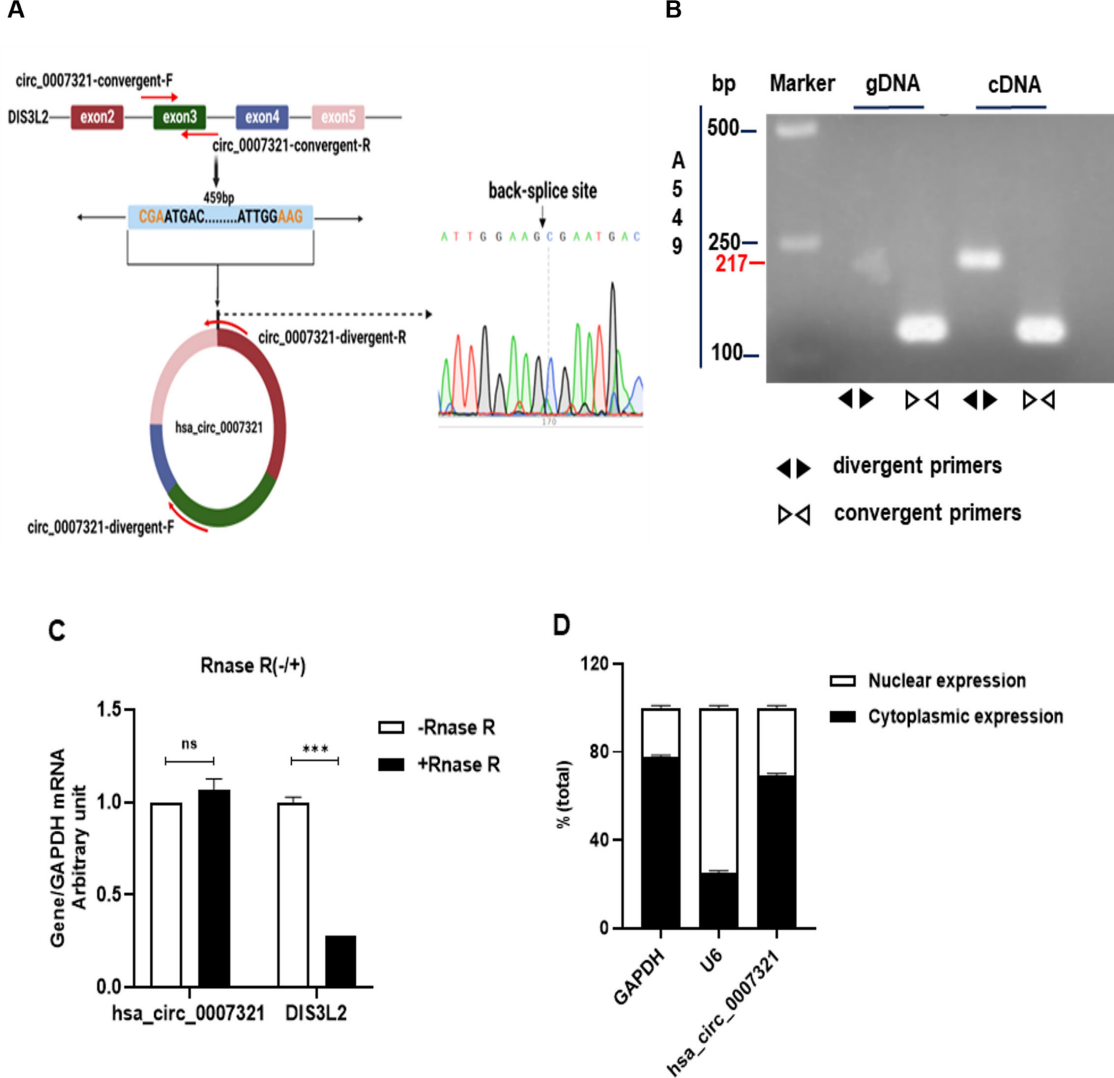
Characterization of hsa_circ_0007321. (**A**) Schematic illustration showing the genomic region of hsa_circ_0007321 derived from exons 2, 3, 4, and 5 of the DIS3L2 gene. Convergent and divergent primers were designed to amplify the linear or back-splicing products. The back-spliced junction site of hsa_circ_0007321 was confirmed by Sanger sequencing. (**B**) Hsa_circ_0007321 and DIS3L2 from cDNA and gDNA amplified by divergent and convergent primers were detected by agarose gel electrophoresis. Divergent primers amplified hsa_circ_0007321 in cDNA but not in gDNA. (**C**) Total RNAs from A549 cells with or without RNase R treatment were subject to qRT-PCR to determine hsa_circ_0007321 and DIS3L2 mRNA expression. Hsa_circ_0007321 was resistant to RNase R treatment. (**D**) The nucleus and cytoplasm RNA of A549 cells were extracted, and hsa_circ_0007321 and DIS3L2 mRNA expression were determined by qRT-PCR. GAPDH and U6 served as internal references of the cytoplasm and nucleus, respectively. Hsa_circ_0007321 was predominantly located in the cytoplasm of A549 cells. ns represents *P* > 0.05; ***P < 0.001.

Hsa_circ_0007321 consists of exons 2, 3, 4, and 5 of the DIS3L2 gene, with a total length of 459 bp (Fig. 3A). The back-spliced junction site of hsa_circ_0007321 was amplified by divergent primers, and the PCR product was confirmed by Sanger sequencing. As shown in Fig. 3B, divergent primers amplified hsa_circ_0007321 in cDNA but not in genomic DNA (gDNA). In addition, when compared to the linear DIS3L2 mRNA, hsa_circ_0007321 was significantly resistant to RNase R, a 3'- to 5'-exoribonuclease that digests linear RNAs (Fig. 3C). Nucleus-plasma separation assays showed that hsa_circ_0007321 is predominantly localized to the cytoplasm (Fig. 3D). All these data indicate that hsa_circ_0007321 is a cytoplasmic circRNA.

### Hsa_circ_0007321 regulates ZIKV replication

**Fig 4:**
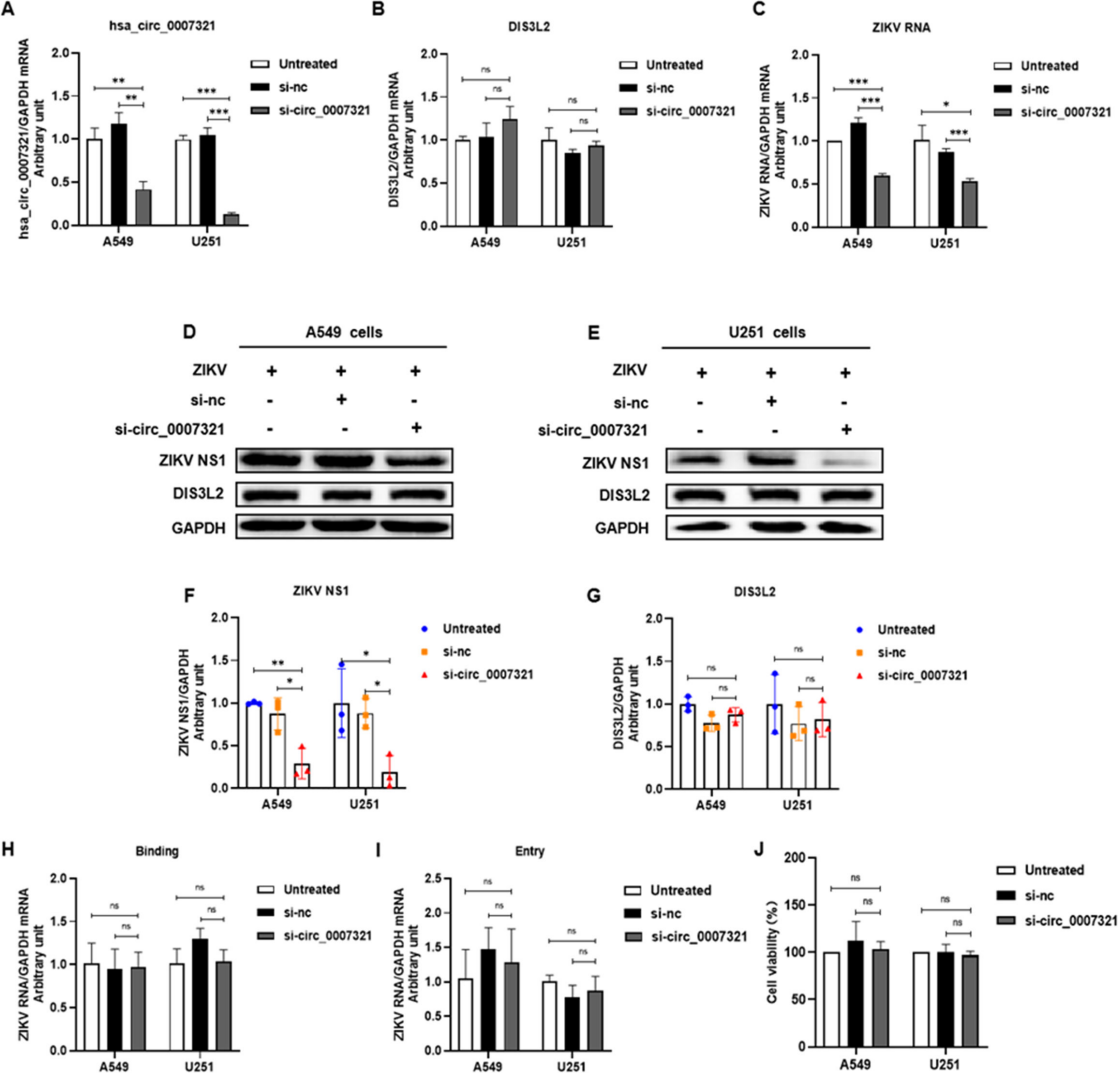
Knockdown of hsa_circ_0007321 inhibited ZIKV replication in A549 and U251 cells. A549 or U251 cells were transfected with si-circ_0007321 or the corresponding negative control siRNA and were infected with ZIKV by MOI of 0.5 at 24 h post-transfection. Total RNAs and proteins in the cells were extracted to detect ZIKV binding, entry, and replication as described in Materials and Methods. ns represent *P* > 0.05; *P < 0.05; **P < 0.01; ***P < 0.001. (**A**) si-circ_0007321 effectively knocked down hsa_circ_0007321 levels in ZIKV-infected A549 and U251 cells. (**B**) si-circ_0007321 did not affect the mRNA level of the hsa_circ_0007321 parental gene DIS3L2. (**C**) Knockdown of hsa_circ_0007321 significantly inhibited ZIKV RNA replication in A549 and U251 cells. (**D and E**) Knockdown of hsa_circ_0007321 inhibited ZIKV NS1 protein expression in A549 and U251 cells without affecting the expression of DIS3L2. (**F and G**) The quantification of ZIKV NS1 and DIS3L2 protein by Image J in A549 (**D**) and U251 (**E**) cells. (**H**) Knockdown of hsa_circ_0007321 did not affect the binding activity of ZIKV. (**I**) Knockdown of hsa_circ_0007321 did not affect the ZIKV entry. (**J**) Knockdown of si-circ_0007321 did not affect cell viability of ZIKV-infected A549 and U251 cells.

Next, we investigated the effect of hsa_circ_0007321 on ZIKV infection and replication. We transfected cells with hsa_circ_0007321 siRNA (si-circ_0007321) or the corresponding negative siRNA (si-nc) for 24 h and then infected them with ZIKV by MOI of 0.5. The cells were incubated for 4 h in a 37°C, 5% CO_2_ incubator and were washed with phosphate-buffered saline (PBS) three times before replacement with fresh culture medium. Total RNA and protein from the cells were collected 48 h later, and the selected gene, ZIKV RNA, and NS1 protein were detected and normalized to GAPDH. We also performed a quantitative analysis for ZIKV NS1 using Image J. We found that hsa_circ_0007321 was significantly knocked down by siRNA (si-circ_0007321) without affecting the expression level of linear DIS3L2 at the mRNA and protein level (Fig. 4A, B, D, and E). Compared with the negative control group, si-circ_0007321 significantly inhibited ZIKV RNA replication to 49.2% ± 1.8% and 61.8% ± 5.3% and decreased NS1 protein expression to 32.2% ± 12% and 25% ± 22.9%, respectively, in A549 and U251 cells (Fig. 4C through G). To explore whether knockdown of hsa_circ_0007321 also affects ZIKV infection, we detected the effect of si-circ_0007321 on ZIKV binding and entry. Cells were transfected with si-circ_0007321 for 24 h and were then infected with ZIKV by MOI of 0.5. Cells were then incubated either at 4°C for 1 h for the binding assay or at 37°C for 1 h for the entry assay. We found that knockdown of hsa_circ_0007321 did not significantly affect binding (Fig. 4H) or entry (Fig. 4I) of ZIKV. We also confirmed that hsa_circ_0007321 knockdown did not affect A549 and U251 cell viability (Fig. 4J). All these findings suggest that knockdown of hsa_circ_0007321 inhibits ZIKV replication while not disrupting ZIKV binding and entry.

### Hsa_circ_0007321 interacts with miR-492

**Fig 5:**
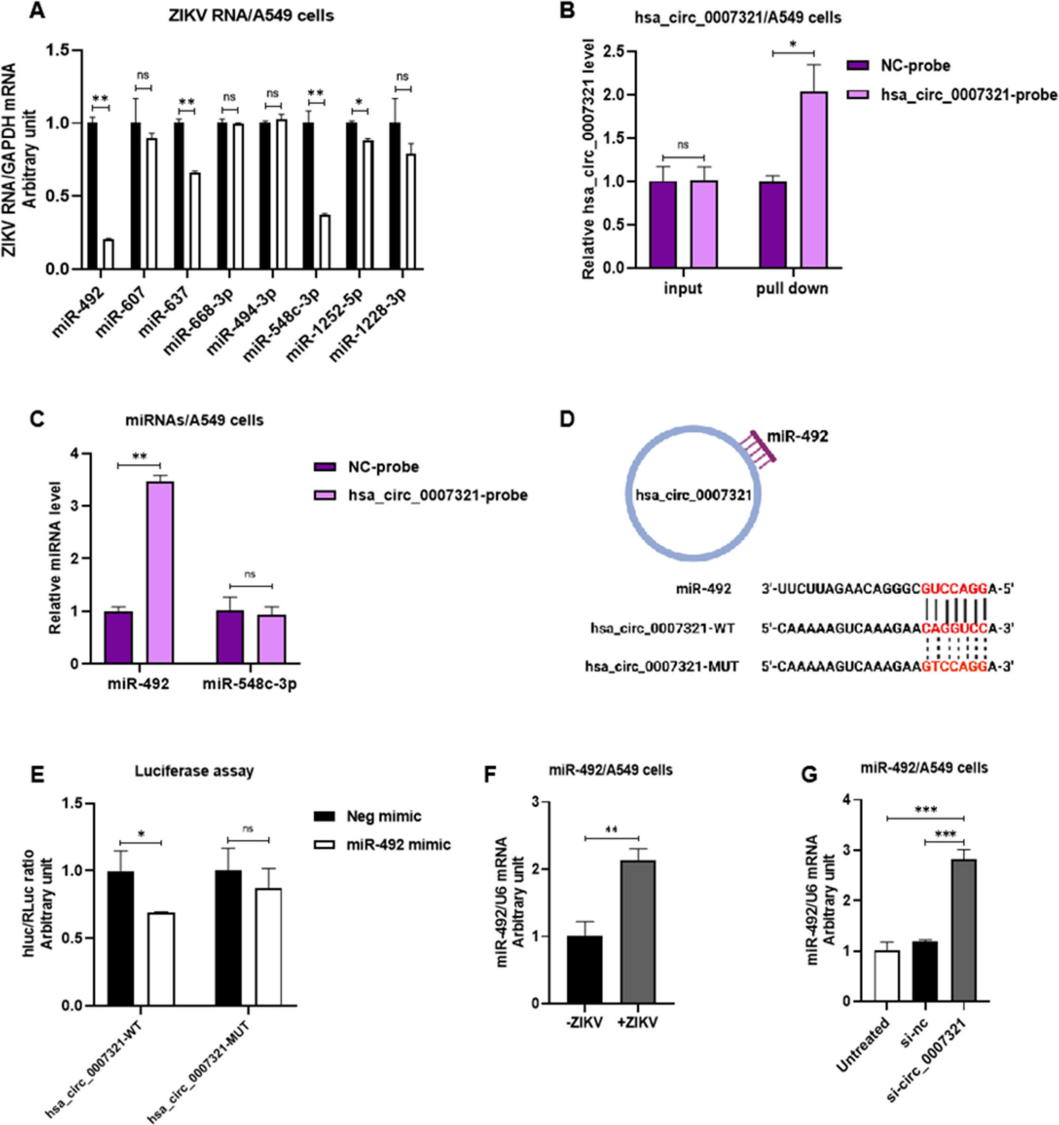
Hsa_circ_0007321 acts as a sponge for miR-492. (**A**) The eight putative target miRNAs with high prediction scores were selected, and the mimics were transfected into A549 cells for 24 h. Then, the cells were infected with ZIKV, and total RNAs were extracted at 48 h post-infection for ZIKV RNA detection. Overexpression of miR-492 and miR-548c-3p significantly inhibited ZIKV RNA replication more profoundly than other miRNAs. (**B and C**) Hsa_circ_0007321 pull down assay. A549 cells were seeded in a 10-cm dish with 90% confluence and were washed with PBS containing RNase inhibitor twice. Then, cells were lysed and incubated with 3 µL of a 100-µM biotin-labeled hsa_circ_0007321 probe or a negative control probe at 37°C for 3 h, respectively. The levels of hsa_circ_0007321, miR-492, and miR-548c-3p were detected using qRT-PCR. (**B**) hsa_circ_0007321 in A549 lysates was pulled down with biotin-labeled hsa_circ_0007321-specific probe. (**C**) MiR-492, but not miR-548c-3p, was significantly enriched by the biotinylated probe against hsa_circ_0007321. (**D**) Schematic of the complementary sequence between hsa_circ_0007321 and miR-492. (**E**) The full-length hsa_circ_0007321 was constructed into a dual-luciferase reporter gene vector pmirGLO to obtain pmirGLO-circ_0007321 WT. The pmirGLO-circ_0007321-MUT plasmid was constructed based on the WT plasmid with a 5′-CAGGTCC-3′ to 5′-GTCCAGG-3′ mutation at the binding site. The reporter plasmids were co-transfected into 293T cells with miR-492 mimic or the corresponding negative control. MiR-492 decreased firefly*/Renilla* luciferase activity when co-transfected with pmiRGLO-circ_0007321 WT. (**F**) A549 cells were infected with ZIKV at an MOI of 0.5. RNAs were extracted, and miR-492 was tested using qRT-PCR at 48 h post-infection. MiR-492 was significantly upregulated in ZIKV-infected A549 cells compared to uninfected cells. (**G**) A549 cells were transfected with hsa_circ_0007321 siRNA or the corresponding negative control and infected with ZIKV at an MOI of 0.5. Total RNAs were harvested, and miR-492 expression was detected at 48 h post-infection. Knockdown of hsa_cric_0007321 increased miR-492 expression. ns represent *P* >0.05; *P < 0.05; **P < 0.01; ***P < 0.001.

As hsa_circ_0007321 was abundant in cytoplasm, we therefore hypothesized that hsa_circ_0007321 may act as a ceRNA for miRNAs. We predicted the potential target miRNAs of hsa_circ_0007321 using the database CircInteractome. A total of 21 miRNAs were predicted to have at least one binding site with hsa_circ_0007321. Among these miRNAs, miR-492 has the highest prediction score. We selected eight putative target miRNAs with high prediction scores by bioinformatics (CircInteractome) and tested their effects on ZIKV replication. We found that overexpression of miR-492 and miR-548c-3p significantly reduced ZIKV replication to 20.5% ± 1.1% and 37.5% ± 2.5%, respectively, when compared to other miRNAs (miR-607, 90.5% ± 12.8%; miR-637, 66.5% ± 1.8%; miR-668-3p, 99.5% ± 13.5%; miR-494-3p, 102.5% ± 1.5%; miR-1252-5p, 88% ± 1.9%; and miR-1228-3p, 80.2% ± 14.2%) in A549 cells (Fig. 5A). We then performed an RNA pull-down assay using a biotin-coupled hsa_circ_0007321 probe to investigate the interaction between hsa_circ_0007321 and miR-492 and miR-548c-3p. We found that miR-492 was significantly enriched in the hsa_circ_0007321 probe group compared to the negative control group and miR-548c-3p group. These findings suggest specific interactions between miR-492 and hsa_circ_0007321 (Fig. 5B and C). To further confirm whether miR-492 is a direct target of hsa_circ_0007321, we performed a dual-luciferase assay. MiR-492 mimic was co-transfected into the cells with either pmiRGLO-circ_0007321-WT-Luc (expression firefly luciferase) or pmiRGLO-circ_0007321-MUT-Luc (expression firefly luciferase) and pRL-TK (expressing *Renilla* luciferase as internal control) (Fig. 5D). We found that miR-492 significantly reduced the firefly*/Renilla* luciferase activity of pmiRGLO-circ_0007321-WT-Luc (~31%) but not pmiRGLO-circ_0007321-MUT-Luc (Fig. 5E). These findings indicate that miR-492 is a direct target of hsa_circ_0007321. Moreover, we found that miR-492 was enriched after ZIKV infection (Fig. 5F) in contrast to the downregulation of hsa_circ_0007321 by ZIKV infection. Knockdown of hsa_circ_0007321 significantly increased miR-492 levels by 2.37 ± 0.17-fold in A549 cells (Fig. 5G). Together these results indicate that ZIKV-induced hsa_circ_0007321 suppression activates miR-492 to exert its regulatory function.

### Hsa_circ_0007321/miR-492 inhibits ZIKV replication through activation of the NF-κB signaling pathway

**Fig 6:**
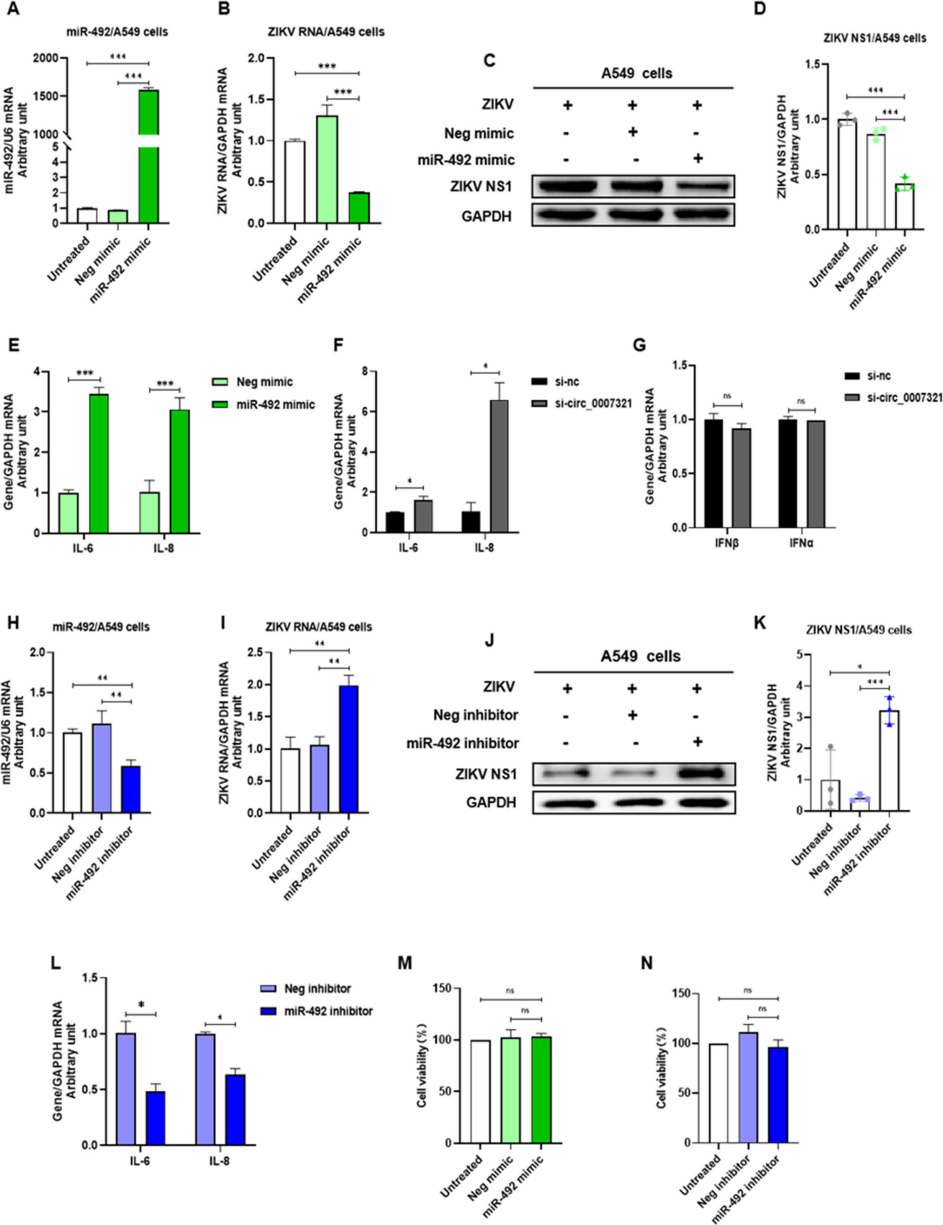
Hsa_circ_0007321/miR-492 regulates ZIKV replication and proinflammatory factor expression in ZIKV-infected A549 cells. A549 cells were transfected with miR-492 mimic, miR-492 inhibitor, hsa_circ_0007321 siRNA, or their corresponding negative control, respectively, and were infected with ZIKV at an MOI of 0.5. Total RNAs and proteins were harvested at 48  h post-infection. Selected gene expression levels were detected by qRT-PCR and Western blot. The densitometry of each blot was analyzed by Image J software. ns represents *P* > 0.05; *P < 0.05; **P < 0.01; ***P < 0.001. (**A**) MiR-492 mimic increased miR-492 expression levels significantly in ZIKV-infected A549 cells. (**B**) Overexpression of miR-492 inhibited ZIKV RNA replication. (**C**) Overexpression of miR-492 inhibited ZIKV NS1 protein levels. (**D**) Quantification of ZIKV NS1 in Fig. 6C by Image J. (**E**) Overexpression of miR-492 increased expression of inflammatory cytokines IL-6 and IL-8 in ZIKV-infected A549 cells. (**F**) Knockdown of hsa_circ_0007321 increased mRNA expression of inflammatory cytokines IL-6 and IL-8 in ZIKV-infected A549 cells. (**G**) Knockdown of hsa_circ_0007321 had no effect on IFNα/β expression. (**H**) MiR-492 inhibitor significantly reduced the expression of miR-492. (**I**) MiR-492 inhibitor promoted ZIKV RNA replication in A549 cells. (**J**) MiR-492 inhibitor promoted ZIKV NS1 protein expression in A549 cells. (**K**) Quantification of ZIKV NS1 in Fig. 6J by Image J. (**L**) MiR-492 inhibitor decreased the mRNA levels of inflammatory cytokines IL-6 and IL-8 in ZIKV-infected A549 cells. (**M and N**) miR-492 mimic and miR-492 inhibitor did not affect viability of ZIKV-infected A549 cells.

Next, we explored the regulatory role of miR-492 on ZIKV replication. MiR-492 was successfully overexpressed after mimic transfection (Fig. 6A). Overexpression of miR-492 significantly reduced ZIKV RNA replication to 29% ± 2.1% (Fig. 6B) and NS1 protein expression to 47.9% ± 4.7% (Fig. 6C and D), which was consistent with the results from hsa_circ_0007321 knockdown (Fig. 4C through F). We also found that miR-492 overexpression and hsa_circ_0007321 knockdown increased the expression of inflammatory cytokines IL-6 and IL-8 in ZIKV-infected A549 cells (Fig. 6E and F). Furthermore, knockdown of hsa_circ_0007321 did not significantly affect the expression of type I interferons (IFNα/β) (Fig. 6G). In contrast, treatment with an miR-492 inhibitor enhanced ZIKV RNA replication by 1.90 ± 0.28-fold (Fig. 6I) and NS1 protein expression by 8.04 ± 1.52-fold (Fig. 6J and K) while decreasing expression of IL-6 and IL-8 (Fig. 6L). Both miR-492 mimic and inhibitor transfection did not significantly affect cell viability (Fig. 6M and N). These findings suggested that knockdown of hsa_circ_0007321 increased miR-492, leading to the activation of antiviral inflammatory cytokines production and, subsequently, ZIKV replication inhibition.

**Fig 7:**
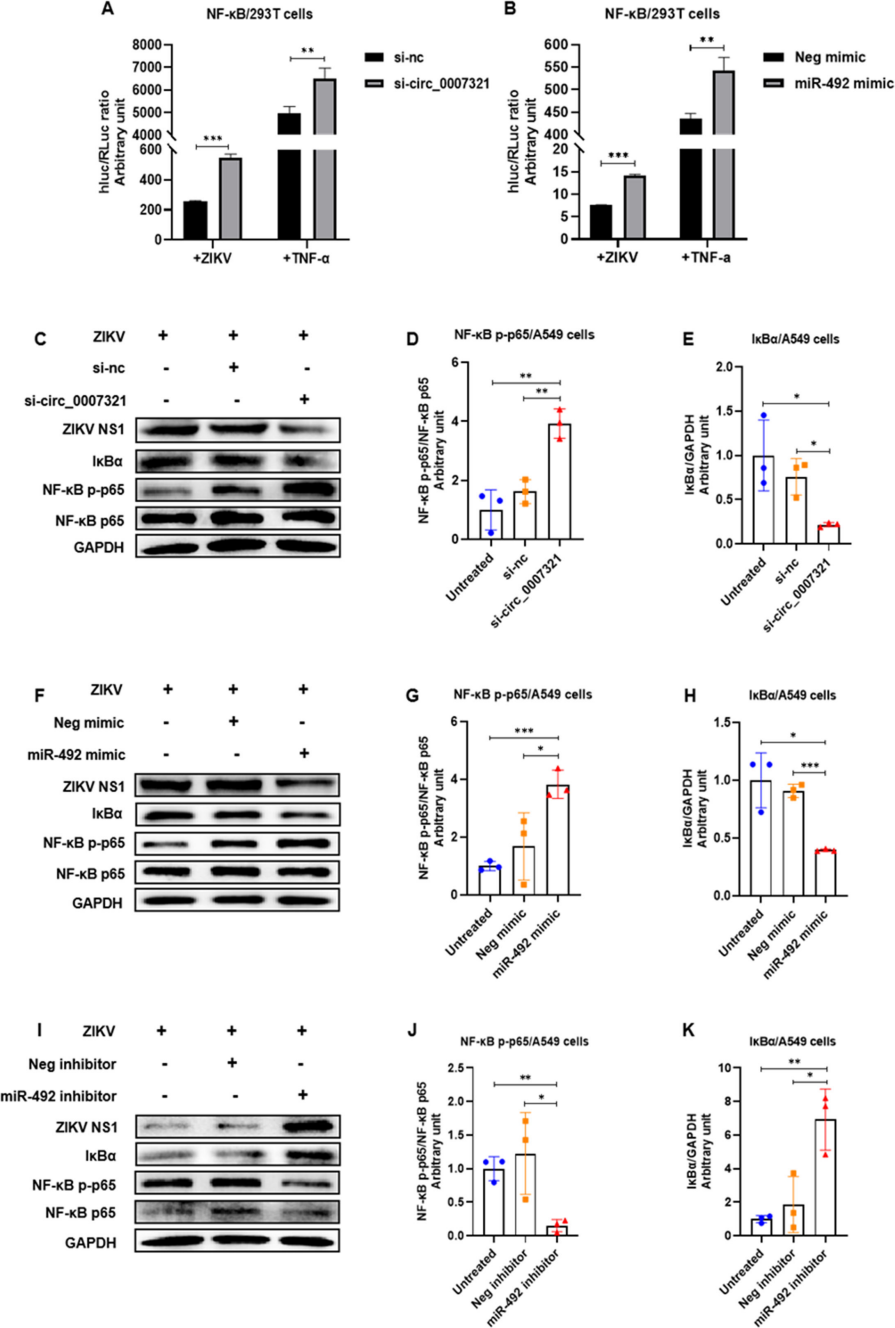
Hsa_circ_0007321/miR-492 regulates ZIKV replication through activating the NF-κB signaling pathway. *P < 0.05; **P < 0.01; ***P < 0.001. (**A and B**) 293T cells were co-transfected with the NF-κB luciferase reporter plasmids (pNF-κB-Luc) and si-circ_0007321, or miR-492 mimic, or their corresponding negative control. Then cells were infected with ZIKV or treated with TNF-α for 12 h. Cell lysates were then prepared for luciferase assays. Relative luciferase activities were obtained by normalizing the *firefly* luciferase to *Renilla* luciferase activities. (**A**) Knockdown of hsa_circ_0007321 increased NF-κB promoter activity in ZIKV-infected and TNF-α-treated 293T cells. (**B**) Overexpression of miR-492 increased NF-κB promoter activity in ZIKV-infected and TNF-α-treated 293T cells. (**C–K**) A549 cells were transfected with miR-492 mimic, si-circ_0007321, miR-492 inhibitor, or their corresponding negative control. ZIKV NS1, total IκBα, NF-κB p65, and phosphorylated NF-κB p65 protein levels were detected by Western blot. The densitometry of each blot was analyzed by Image J software. (**C**) Knockdown of hsa_circ_0007321 significantly decreased total IκBα protein and increased phosphorylation of NF-κB p65. (**D and E**) Quantification of NF-κB p-p65 (**D**) and IκBα (**E**) in Fig. 7C by Image J. (**F**) Overexpression of miR-492 decreased total IκBα protein and significantly increased phosphorylation of NF-κB p65. (**G and H**) Quantification of NF-κB p-p65 (**G**) and IκBα (**H**) in Fig. 7F by Image J. (**I**) MiR-492 inhibitor increased total IκBα protein and decreased phosphorylation of NF-κB p65. (**J and K**) Quantification of NF-κB p-p65 (**J**) and IκBα (**K**) in Fig. 7I by Image J.

As NF-κB is a pivotal mediator of the inflammatory response, we therefore inquired whether the NF-κB signaling pathway was regulated by hsa_circ_0007321/miR-492. We co-transfected the luciferase reporter gene plasmids pNF-κB-luc (containing the NF-κB promoter and expression firefly luciferase) and pRL-TK (expression *Renilla* luciferase as internal control) in addition to either si-circ_0007321 or miR-492 mimic into 293T cells. We found that hsa_circ_0007321 siRNA and miR-492 mimic treatment each significantly increased ZIKV-induced NF-κB relative luciferase activity in 293T cells. Hsa_circ_0007321 siRNA increased ZIKV-induced NF-κB relative luciferase activity by 2.13 ± 0.10-fold compared to the control group, while miR-492 mimic increased ZIKV-induced NF-κB relative luciferase activity by 1.88 ± 0.04-fold. TNF-α stimulation further increased ZIKV infection, and si-circ_0007321 or miR-492 mimic treatment induced NF-κB relative luciferase activity in 293T cells (Fig. 7A and B). These findings indicated that hsa_circ_0007321 knockdown or miR-492 overexpression induced activation of the NF-κB signaling pathway. We also examined the effect of hsa_circ_0007321/miR-492 on IκBα and NF-κB p65 phosphorylation. We found that hsa_circ_0007321 knockdown and miR-492 overexpression significantly reduced total protein levels of IκBα and increased phosphorylation of NF-κB p65 (Fig. 7C through H). IκBα expression normally is not affected by miR-492 inhibitor in the absence of any signal as its promoter is controlled in feedback. The observations that miR-492 inhibitor treatment increased total IκBα protein and inhibited NF-κB p65 phosphorylation compared to the control are likely due to the system and the use of inhibitors (Fig. 7I through K). These findings indicated that IκBα degradation and NF-κB p65 phosphorylation are critical steps for ZIKV infection and that si-circ_0007321 or miR-492 mimic treatment can active NF-κB.

**Fig 8:**
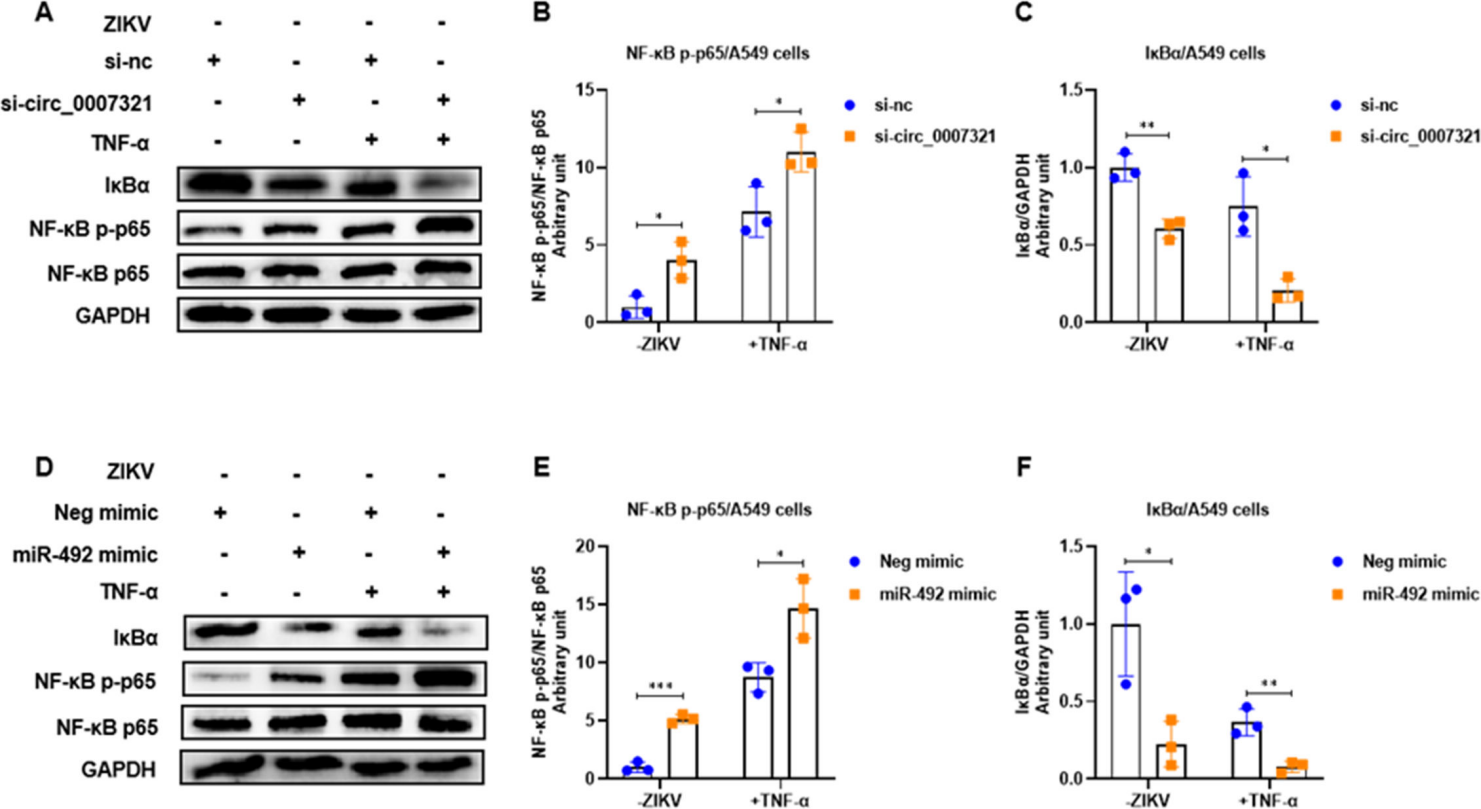
Knockdown of hsa_circ_0007321 and overexpression of miR-492 activate the NF-κB signaling pathway with or without TNF-α stimulation. A549 cells were transfected with miR-492 mimic, si-circ_0007321, or their corresponding negative control. TNF-α was added with a final concentration of 25 ng/mL as a positive control for 12 h. Total IκBα, NF-κB p65, and phosphorylated NF-κB p65 protein levels were detected by Western blot. The densitometry of each blot was analyzed by Image J software. *P < 0.05; **P < 0.01; ***P < 0.001. (**A**) Knockdown of hsa_circ_0007321 and TNF-α stimulation reduced IκBα protein and promoted NF-κB p65 phosphorylation in A549 cells in a cooperative manner. (**B and C**) Quantification of NF-κB p-p65 (**B**) and IκBα (**C**) in Fig. 8A by Image J. (**D**) Overexpression of miR-492 and TNF-α stimulation reduced IκBα protein and promoted NF-κB p65 phosphorylation in A549 cells in a cooperative manner. (**E and F**) Quantification of NF-κB p-p65 (**E**) and IκBα (**F**) in Fig. 8D by Image J.

**Fig 9:**
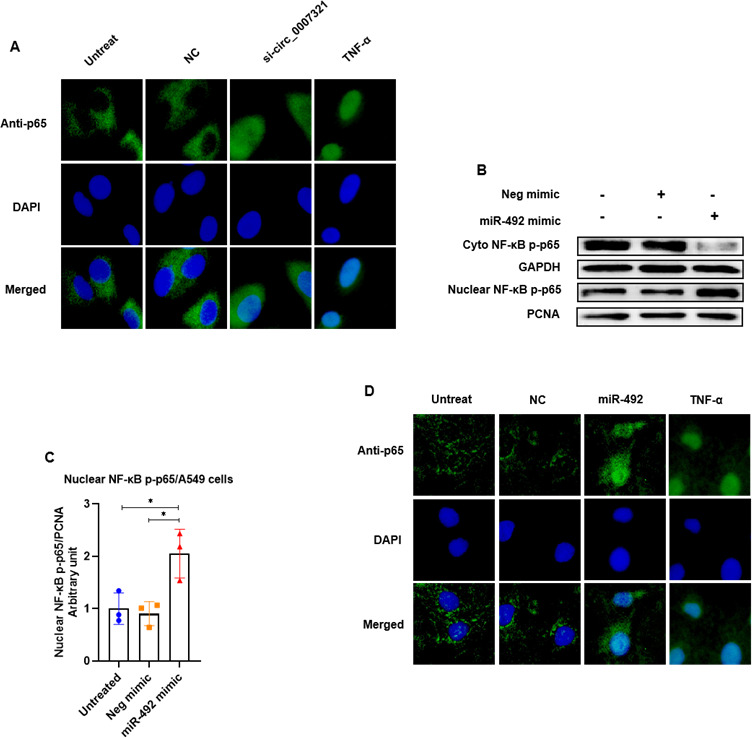
Hsa_circ_0007321/miR-492 activates the NF-κB pathway by promoting p65 nuclear translocation in A549 cells. (**A**) A549 cells were transfected with si-circ_0007321 or the negative control. TNF-α treatment was used as a positive control. Cells were fixed and stained with fluorescent probes for DNA (DAPI; blue) and NF-κB p65 (green). Knockdown of hsa_circ_0007321 promoted NF-κB p65 nuclear translocation. (**B and C**) A549 cells were transfected with miR-492 mimic or negative mimic control. The cytoplasmic and nuclear proteins were isolated, and NF-κB p-p65 levels were detected using Western blot. GAPDH and proliferating cell nuclear antigen (PCNA) were used as a loading control for cytoplasmic and nuclear protein, respectively. The densitometry of each blot was analyzed by Image J software. (**B**) Overexpression of miR-492 decreased NF-κB p-p65 levels in the cytoplasm while increasing NF-κB p-p65 levels in the nucleus. (**C**) Quantification of NF-κB p-p65 in Fig. 9B by Image J. (**D**) A549 cells were transfected with miR-492 mimic or negative mimic control. TNF-α treatment was used as a positive control. Cells were fixed and stained with fluorescent probes for DNA (DAPI; blue) and NF-κB p65 (green). Overexpression of miR-492 promoted NF-κB p65 nuclear translocation. *P < 0.05.

We also confirmed the effect of hsa_circ_0007321 and miR-492 on NF-κB activation in the absence of ZIKV infection. TNF-α treatment was used as a positive control. We confirmed that hsa_circ_0007321 knockdown and miR-492 overexpression significantly reduced total protein expression of IκBα and promoted phosphorylation of NF-κB p65 in A549 cells both with and without TNF-α treatment (Fig. 8). Moreover, we observed that hsa_circ_0007321 knockdown and miR-492 overexpression enhanced NF-κB p65 nuclear translocation in A549 cells (Fig. 9). These data suggest that knockdown of hsa_circ_0007321 increases miR-492, which then activates the NF-κB signaling pathway leading to the production of inflammation cytokines.

### Hsa_circ_0007321/miR-492 targets NFKBID to activate NF-κB

**Fig 10:**
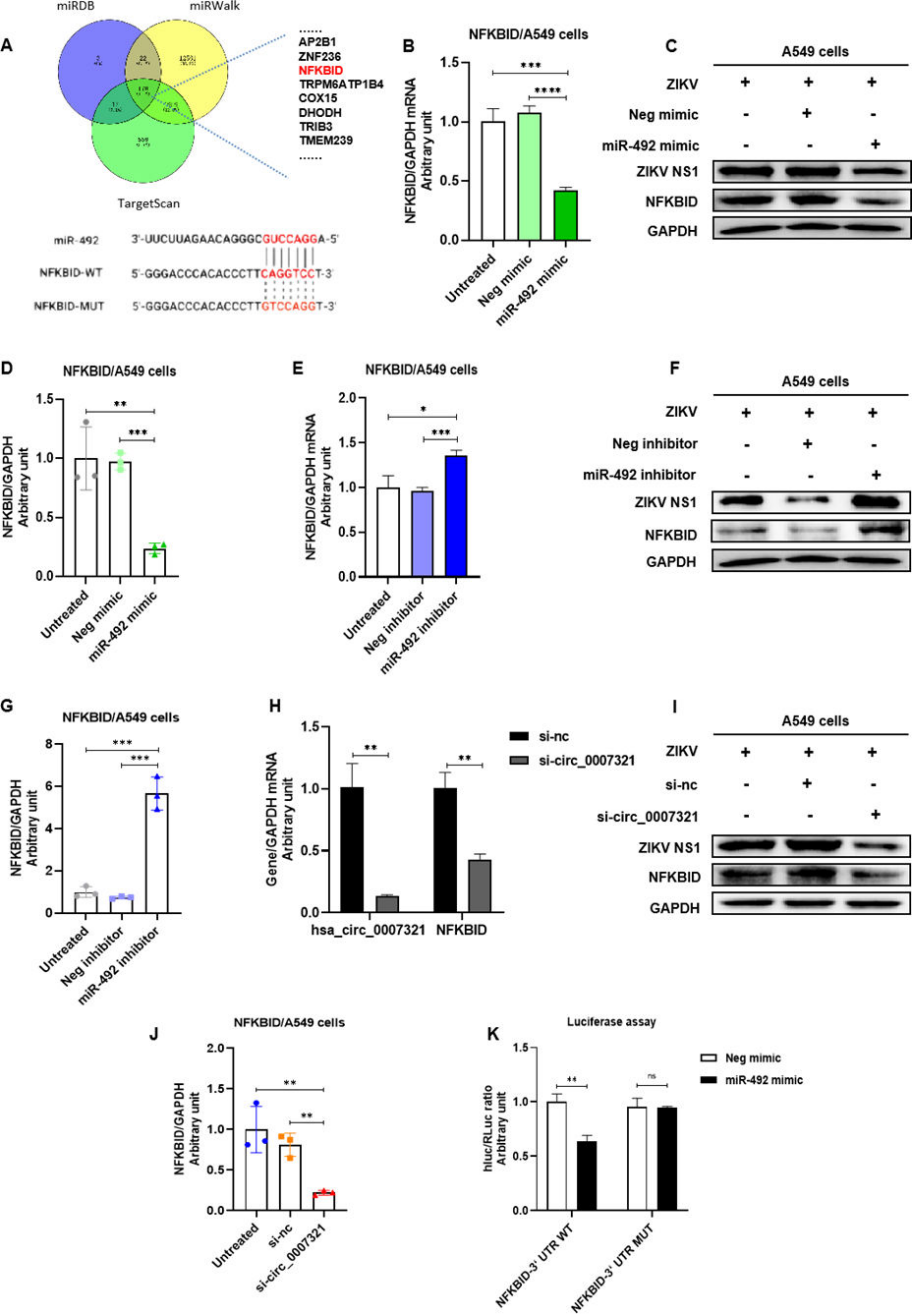
Hsa_circ_0007321/miR-492 regulates NFKBID expression by directly targeting its 3*'* UTR. (**A**) The potential target genes of miR-492 were predicted by three algorithms. One possible miR-492-binding site was predicted at the 3*'* UTR region of NFKBID. (**B–D**) A549 cells were transfected with miR-492 mimic or negative mimic control and then infected with ZIKV. Total RNAs and proteins were harvested at 48 h post infection. NFKBID expression was detected by qRT-PCR and Western blot. The densitometry of each blot was analyzed by Image J. (**B**) Overexpression of miR-492 downregulated NFKBID mRNA levels. (**C**) Overexpression of miR-492 downregulated NFKBID protein levels. (**D**) Quantification of NFKBID in Fig. 10C by Image J. (**E and G**) A549 cells were transfected with miR-492 inhibitor or negative inhibitor control and then infected with ZIKV. Total RNAs and proteins were harvested at 48 h post infection. NFKBID expression was detected by qRT-PCR and Western blot. The densitometry of each blot was analyzed by Image J. (**E**)MiR-492 inhibitor upregulated NFKBID mRNA levels. (**F**) MiR-492 inhibitor upregulated NFKBID expression at protein levels. (**G**) Quantification of NFKBID in Fig. 10F by Image J. (**H and J**) A549 cells were transfected with si-circ_0007321 or si-nc and then infected with ZIKV. Total RNAs and proteins were harvested at 48 h post infection. Selected gene expression was detected by qRT-PCR and Western blot. The densitometry of each blot was analyzed by Image J. (**H**) Knockdown of hsa_circ_0007321 decreased NFKBID mRNA levels in A549 cells. (**I**) Knockdown of hsa_circ_0007321 decreased NFKBID protein levels in A549 cells. (**J**) Quantification of NFKBID in Fig. 10I by Image J. (**K**) Wild-type or mutant NFKBID 3′ UTR sequences containing the binding sites of miR-492 seed sequence were constructed into the reporter vector pmirGLO and co-transfected with miR-492 mimic or negative mimic control into 293T cells. Cell lysates were then prepared for luciferase assays. Relative luciferase activities were obtained by normalizing the *firefly* luciferase to *Renilla* luciferase activities. MiR-492 decreased firefly*/Renilla* luciferase activity when co-transfected with pmiRGLO-NFKBID-WT. ns represents *P* > 0.05; *P < 0.05; **P < 0.01; ***P < 0.001.

Next, we investigated the target genes of miR-492. We predicted possible target genes of miR-492 using three algorithms, such as TargetScan, miRDB, and miRWalk. The overlapping genes predicted by all three of these databases were then selected (Fig. 10A). Among these genes, an atypical NF-κB inhibitor gene, NFKBID (IkBδ), was predicted to have one possible miR-492-binding site in the 3*'* UTR region. We found overexpression of miR-492 significantly downregulated NFKBID mRNA expression to 39.2% ± 3.8% and protein levels to 24.2% ± 2.6% (Fig. 10B through D). Conversely, miR-492 inhibitor markedly enhanced NFKBID expression (Fig. 10E and G). We also investigated the effect of hsa_circ_0007321 knockdown on NFKBID expression. Consistent with the effect of miR-492 overexpression, knockdown of hsa_circ_0007321 significantly reduced NFKBID mRNA levels to 42.3% ± 1.9% and protein levels to 27.5% ± 1.6% (Fig. 10H through J). We further verified that NFKBID is a direct target of miR-492 by a dual-luciferase reporter assay. Notably, miR-492 significantly inhibited the firefly*/Renilla* luciferase activity of pmiRGLO-NFKBID-WT-Luc (expressing firefly) and pRL-TK (expressing *Renilla*) but had no effect on the firefly*/Renilla* luciferase activity of pmiRGLO-NFKBID-MUT-Luc (Fig. 10K). These results suggest that NFKBID is a target of miR-492.

### Hsa_circ_0007321/miR-492 regulates ZIKV replication in an NFKBID/NF-κB-dependent manner

**Fig 11:**
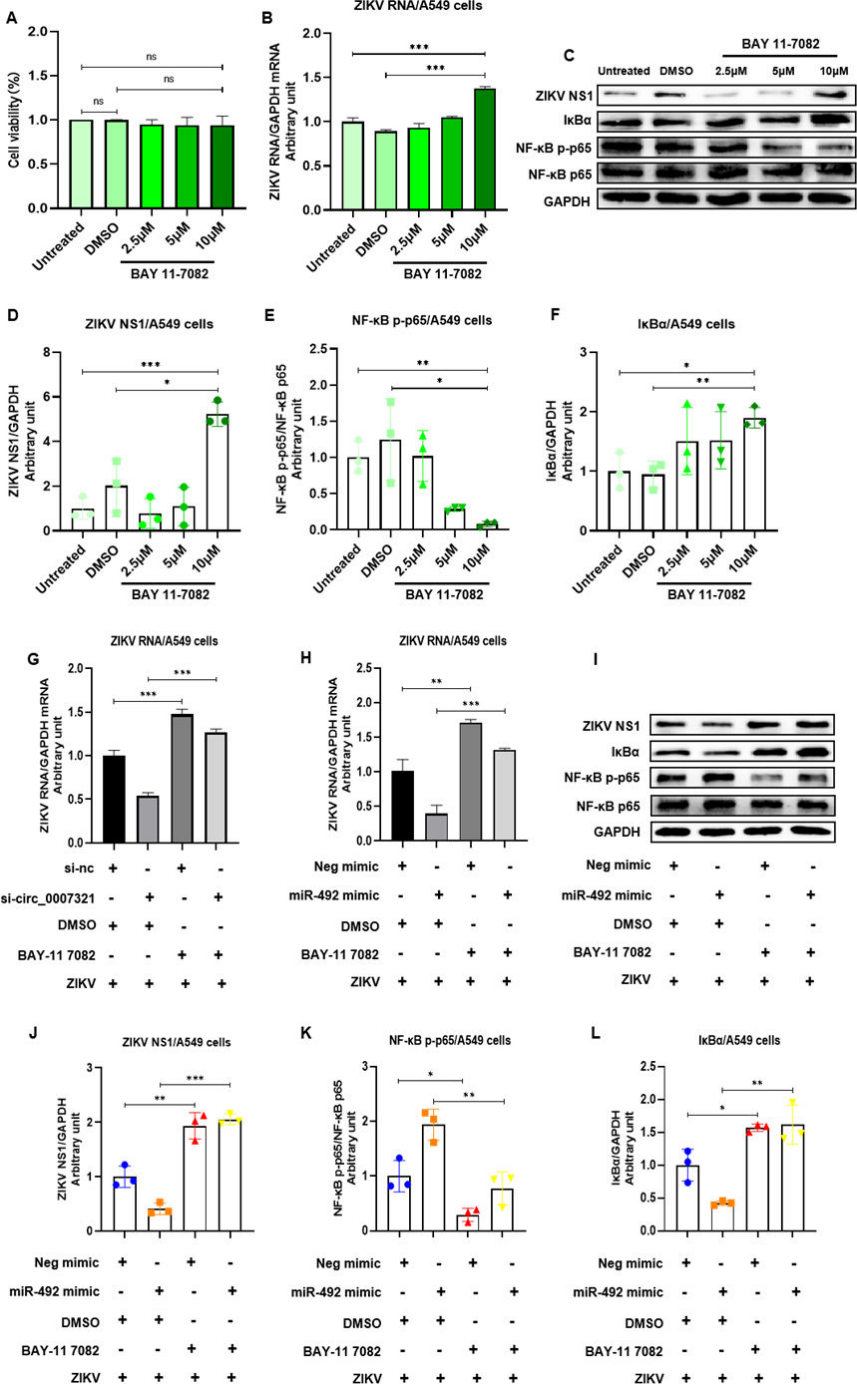
BAY 11-7082 promoted ZIKV replication and abrogated the inhibitory effect of si_circ_0007321 and miR-492 mimic on ZIKV replication. A549 cells were pretreated with BAY 11-7082 for 1 h and were then infected with ZIKV. For combination treatment, miR-492 mimic or si-circ_0007321 was transfected into cells after BAY 11-7082 treatment, followed by ZIKV infection for 48 h. Total RNA and protein were harvested for gene detection by qRT-PCR or Western blot. The densitometry of each blot was analyzed by Image J. (**A**) BAY 11-7082 did not affect cell viability in concentrations up to 10 µM. (**B**) 10 µM BAY 11-7082 treatment increased ZIKV RNA levels significantly. (**C**) 10 µM BAY 11-7082 treatment decreased NF-κB p-p65 levels and increased IκBα and ZIKV NS1 protein levels. (**D–F**) The quantification of ZIKV NS1 (**D**), NF-κB p-p65 (**E**), and IκBα (**F**) in Fig. 11C by Image J. (**G**) Pretreatment with BAY 11-7082 abrogated the effect of hsa_circ_0007321 siRNA on ZIKV RNA replication in A549 cells. (**H**) Pretreatment with BAY 11-7082 abrogated the effect of miR-492 mimic on ZIKV RNA replication in A549 cells. (**I**) Pretreatment with BAY 11-7082 abrogated the effect of miR-492 mimic on the NF-κB pathway. (**J and L**) Quantification of ZIKV NS1 (**J**), NF-κB p-p65 (**K**), and IκBα (**L**) in Fig. 11I by Image J. ns represents *P* > 0.05; *P < 0.05; **P < 0.01; ***P < 0.001.

We then proceeded to explore the role of NFKBID/NF-κB on hsa_circ_0007321/miR-492-mediated regulation of ZIKV replication by blocking the NF-κB pathway with its inhibitor reagent BAY 11-7082 or through knockdown of NFKBID. We found that cells pretreated with 10 µM BAY 11-7082 had significantly increased total IκBα protein, decreased NF-κB p65 phosphorylation, and enhanced ZIKV RNA replication and NS1 protein expression without a significant effect on cell viability (Fig. 11A through F). Then, we transfected si_circ_0007321 or miR-492 mimic into the BAY 11-7082-pretreated cells and found that BAY 11-7082 pretreatment abrogated the inhibitory effect of si_circ_0007321 and miR-492 mimic on ZIKV replication (Fig. 11G through L).

**Fig 12:**
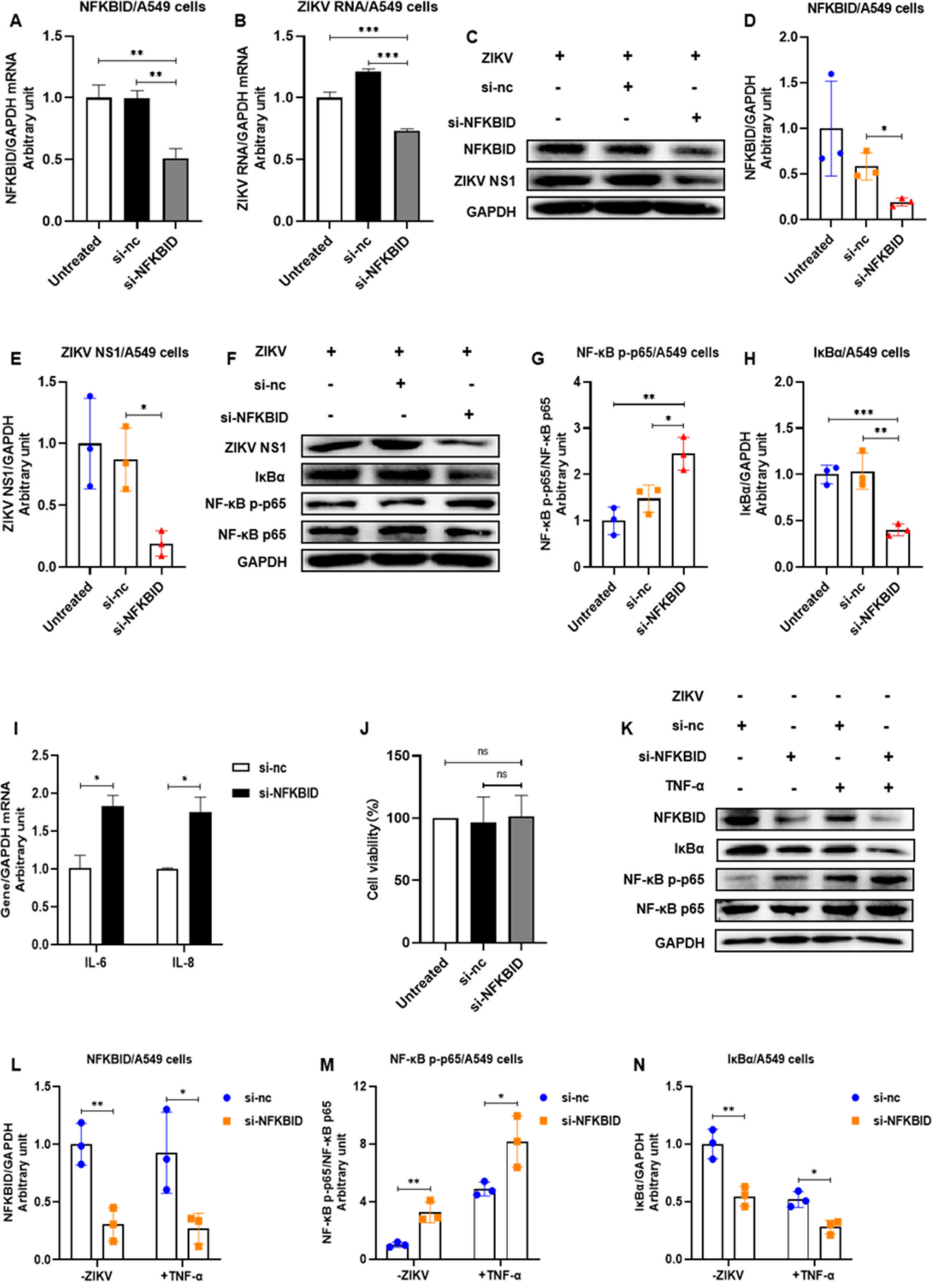
Knockdown of NFKBID inhibited ZIKV replication by activating NF-κB pathway in ZIKV-infected A549 cells. (**A–J**) A549 cells were transfected with si-NFKBID and were then infected with ZIKV for 48 h. Total RNAs and protein were harvested for gene detection by qRT-PCR or Western blot. The densitometry of each blot was analyzed by Image J. (**A**) NFKBID siRNA significantly reduced NFKBID mRNA levels in ZIKV-infected A549 cells. (**B**) Knockdown of NFKBID inhibited ZIKV RNA replication in ZIKV-infected A549 cells. (**C**) Knockdown of NFKBID inhibited protein expression of NFKBID and ZIKV NS1. (**D and E**) Quantification of NFKBID (**D**) and ZIKV NS1 (**E**) in Fig. 12C by Image J. (**F**) Knockdown of NFKBID decreased total IkBa protein and promoted NF-κB p65 phosphorylation in ZIKV-infected A549 cells. (**G and H**) Quantification of NF-κB p-p65 (**G**) and IκBα (**H**) in Fig. 12F by Image J. (**I**) Knockdown of NFKBID increased the mRNA levels of inflammatory cytokines IL6 and IL8. (**J**) si-NFKBID did not affect the viability of A549 cells. (**K–M**) A549 cells were transfected with si-NFKBID. Total RNAs and proteins were harvested for gene detection by qRT-PCR or Western blot. TNF-α treatment at 25 ng/mL was used as a positive control. (**K**) NFKBID silencing similarly reduced IκBα protein and promoted NF-κB p65 phosphorylation in the ZIKV-uninfected and TNF-α-stimulated groups. (**L–N**) Quantification of NFKBID (**L**), NF-κB p-p65 (**M**), and IκBα (**N**) in Fig. 12K by Image J. ns represents *P* > 0.05; *P < 0.05; **P < 0.01; ***P < 0.001.

We then knocked down NFKBID via siRNA in ZIKV-infected A549 cells (Fig. 12A) and found that NFKBID silencing remarkably reduced ZIKV RNA replication and NS1 protein expression (Fig. 12B through E), decreased total IκBα protein, increased phosphorylation of NF-κB p65 (Fig. 12F through H), and promoted the expression of inflammatory cytokines IL6 and IL8 in ZIKV-infected A549 cells (Fig. 12I). NFKBID silencing increased IL-6 1.83 ± 0.12-fold and IL-8 1.75 ± 0.12-fold compared to the control group (Fig. 12I). We also confirmed the activation of NF-κB by NFKBID siRNA in A549 cells without virus infection using TNF-α as a positive control (Fig. 12K through N). These findings indicated that NFKBID knockdown inhibited ZIKV replication through activation of the NF-κB signaling pathway.

**Fig 13:**
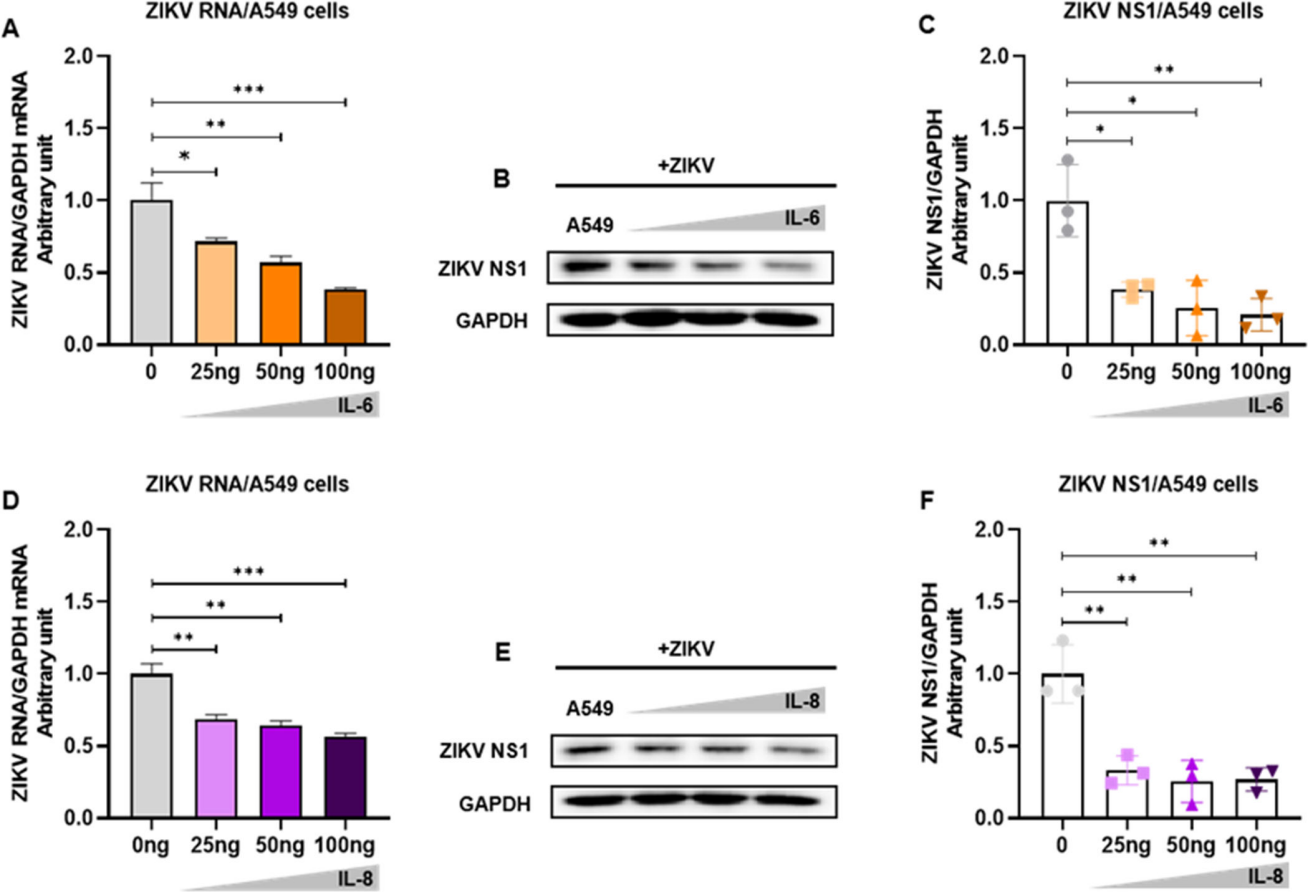
IL-6 and IL-8 inhibited ZIKV replication in the early stages of infection. A549 cells were infected with ZIKV and were treated with either IL-6 or IL-8 at a final concentration of 0, 25, 50, or 100 ng/mL for 48 h. Total RNAs and proteins were harvested for gene detection by qRT-PCR or Western blot. The densitometry of each blot was analyzed by Image J. (**A**) IL-6 treatment inhibits ZIKV RNA replication in a dose-dependent manner. (**B**) IL-6 treatment inhibits ZIKV NS1 expression in a dose-dependent manner. (**C**) The quantification of ZIKV in Fig. 13B by Image J. (**D**) IL-8 treatment inhibits ZIKV RNA replication in a dose-dependent manner. (**E**) IL-8 treatment inhibits ZIKV NS1 expression in a dose-dependent manner. (**F**) The quantification of ZIKV in Fig. 13E by Image J. *P < 0.05; **P < 0.01; ***P < 0.001.

IL-6 has been reported to limit viral replication in the early stages of viral infection (15). Considering that hsa_circ_0007321/miR-492/NFKBID can promote the expression of IL-6 and IL-8, we therefore explored the effects of IL-6 and IL-8 on ZIKV replication. We treated the cells at 24 h post ZIKV infection with IL6 or IL8 at a final concentration of 0, 25, 50, and 100 ng/mL. After 48 h of treatment, total RNA and protein were extracted, and ZIKV replication was measured. We confirmed that both IL-6 and IL-8 inhibited ZIKV RNA replication and NS1 protein expression significantly (Fig. 13). Then, 100 ng/mL of IL-6 reduced ZIKV RNA by ~62.3%, and 100 ng/mL of IL-8 reduced ZIKV RNA by ~43.7%.

## DISCUSSION

In recent years, circRNAs have been reported to play important roles in viral replication and host antiviral immune responses (16–20). There are some studies that have shown that viruses utilize circRNAs to benefit their replication. Shi et al. found that the intracellular circ_0050463 levels were upregulated after influenza A virus (IAV) infection and that silencing circ_0050463 significantly reduced IAV replication and IAV M1 protein expression (21). Zhang et al. reported that circ_0004812 was upregulated in chronic hepatitis B patients and HBV-infected hepatoma cells. Knockdown of circ_0004812 resulted in a significant decrease of HBsAg, HBeAg levels, and HBV DNA copies, and an increase in IFN-α/β expression (22). Viral replication affects host circRNAs, which in turn regulate their target genes to modify virus replication. For example, Qu et al. reported that influenza virus-infected A549 cells have elevated circRNA-AIVR levels which downregulate antiviral target gene AIVR to promote influenza virus replication (23). In contrast, Kaposi’s sarcoma herpesvirus (KSHV) activated hsa_circ_0001400 which inhibits KSHV lytic transcription and blocks virus production (24). In the present study, we found that ZIKV infection decreased hsa_circ_0007321 and subsequently upregulated the NF-κB signaling pathway to inhibit ZIKV replication.

There is growing body of evidence supporting the role of circRNAs as miRNA sponges to upregulate target gene expression and, therefore, regulate viral replication and host antiviral immune responses (23, 25–27). For example, in influenza virus-infected A549 cells, circRNA AIVR was upregulated and promoted the production of IFN-β via sponge-adsorbing miR-330-3p and upregulation of CREBBP to exert its antiviral effect (23). CircPDCD4 regulates porcine circovirus type 2 infection in porcine kidney 15 cells by acting as a miR-21 sponge (28). In this study, we verified the interaction between hsa_circ_0007321 and miR-492 by RNA pull-down and dual-luciferase reporter assays. We also discovered that hsa_circ_0007321 negatively regulates miR-492 expression. These findings suggest that hsa_circ_0007321 targets miR-492 by acting as a sponge, preventing it from exerting its silencing effects. However, miR-492 was not screened out in our original RNAseq data, as only a small number of differently expressed miRNAs have been identified (29), and the original RNAseq was not designed for miRNA screen. Furthermore, the possible target miRNAs of hsa_circ_0007321 were confirmed by RT-qPCR with specific miRNA primers.

NF-κB is one of the most well-known mediators in the host immune system against viral infections (30–32). The activation of NF-κB has been found to be associated with the IFN pathway. For example, IRF3, the upstream transcription factor required for the induction of IFNs, has been reported to interact with the p65 subunit of NF-κB directly in the cytoplasm and prevent its nuclear transport (33). We found that knockdown of hsa_circ_0007321 activated the NF-κB pathway but did not affect IFNα/β expression. We, therefore, speculated that there are other participants involved, including those upstream of IFN production that may influence their system.

The NF-κB transcription factor family, including p65 (RelA), RelB, c-Rel, p105/p50, and p100/p52, forms homo- or heterodimers and remains as inactive complexes with inhibitory molecules called IκB proteins. The canonical NF-κB pathway is activated after the degradation of IκBα, which results in nuclear translocation of NF-κB complexes, predominantly the p50/p65 dimer (5). NFKBID, also known as IκBδ and IκBNS, belongs to the nuclear IκB-like family of proteins that includes Bcl-3 and IκBζ. NFKBID has been reported to specifically associate with the p50 subunit of NF-κB, leading to a change in DNA-binding activity and inhibition of IL-6 transcription (34). In addition, p50 in the cytoplasm interacts with chimeric GST-IκBNS fusion proteins (35). Our findings confirmed that knockdown of NFKBID significantly increases IL6 and IL8 expression. We observed that the knockdown of NFKBID decreased total IκBα protein and increased phosphorylation of NF-κB p65. We, therefore, hypothesized that NFKBID interacts with the p50 subunit of NF-κB. Knockdown of NFKBID activated NF-κB and increased the expression of IL6 and IL8, which in turn also promotes NF-kB activation through the downregulation of IkBα and increased phosphorylation of p65. MiR-3473b has been reported to activate the NF-κB signaling pathway in fibroblasts by downregulating NFKBID and inducing the expression of inflammatory cytokines (IL-6, CCL1, CCL2, CCL5, and CXCL2) (8). In this study, we found that hsa_circ_0007321 knockdown or miR-492 overexpression activated the NF-κB pathway as demonstrated by enhanced NF-κB promoter activity, reduced IκBα protein expression, and increased NF-κB p65 phosphorylation and translocation into the nucleus. NFKBID was confirmed as a direct target of miR-492, and knockdown of NFKBID significantly reduced ZIKV replication and total IκBα protein, while increasing phosphorylation of NF-κB p65. The impact of hsa_circ_0007321/miR-492/NFKBID on IκBα may occur through positive feedback of cytokines including IL6 and IL8. BAY 11-7082 is an inhibitor on IκBα phosphorylation and NF-κB expression. Using BAY 11-7082 treatment abrogated the inhibitory effect of si_circ_0007321 and miR-492 mimic on ZIKV replication, indicating that hsa_circ_0007321/miR-492 regulates ZIKV replication through the NF-κB pathway.

Compared with other forms of non-coding RNAs, circRNAs are stable and conserved and are more suitable to be used as new diagnostic molecular biomarkers and therapeutic targets. Several approaches have been developed to target circRNAs *in vivo*. For example, it has been reported that nanoparticles and exosomes can deliver the siRNA targeting the back-splice junction of circRNAs or can deliver circRNA expression plasmid *in vivo* effectively to induce circRNA cleavage or overexpression (36–39). Further investigation on inhibiting ZIKV replication *in vivo* by targeting hsa_circ_0007321 using a nanoparticle- or exosome-mediated method is warranted.

**Fig 14:**
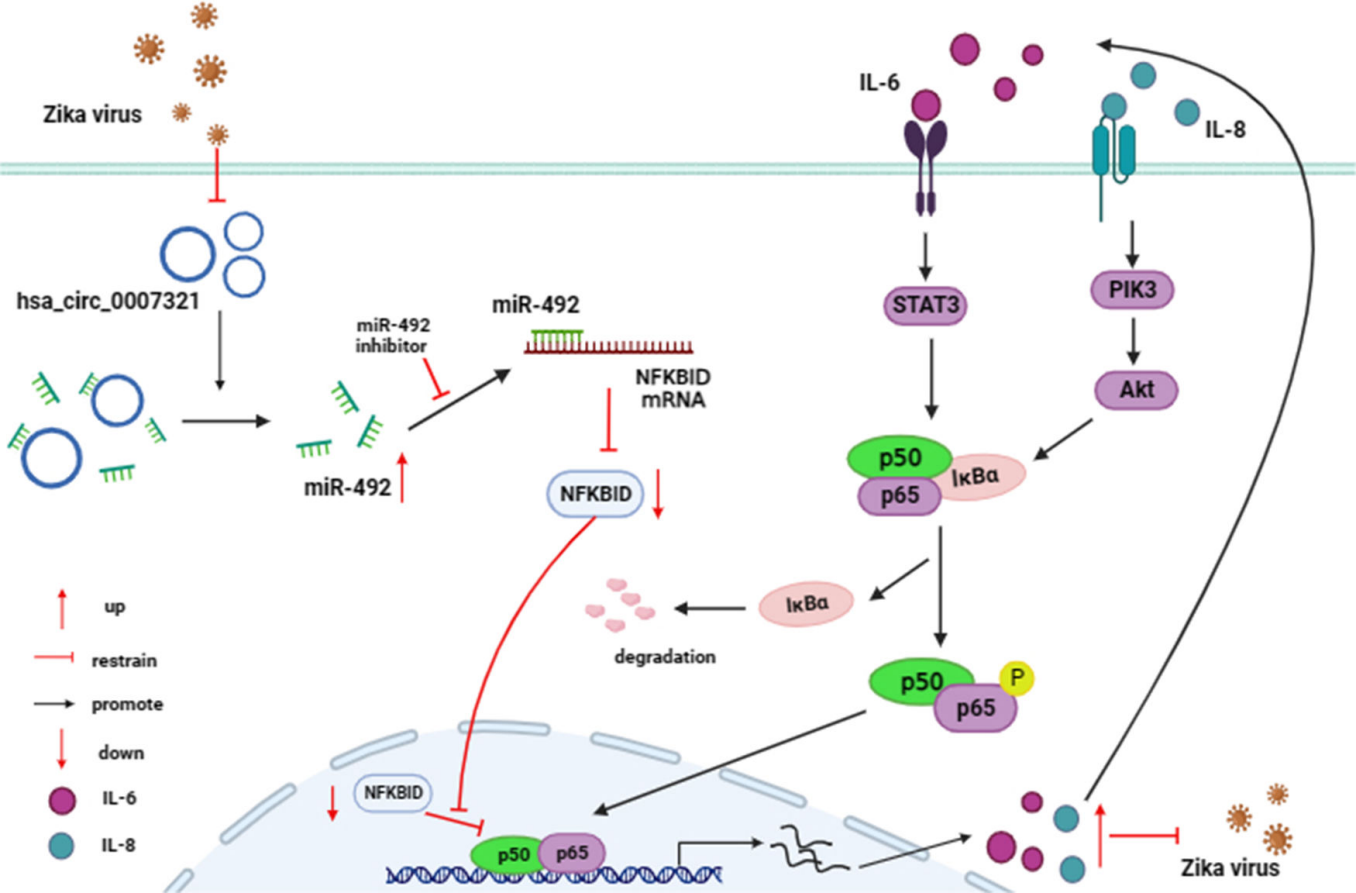
Schematic of the hsa_circ_0007321/miR-492/NFKBID axis regulating ZIKV replication. ZIKV infection decreased hsa_circ_0007321 expression. Downregulation of hsa_circ_000732 increased miR-492 expression as hsa_circ_0007321 acts as an endogenous miR-492 sponge. miR-492 suppressed NFKBID expression by targeting its 3′UTR directly, which led to the activation of the NF-κB signaling pathway and the increased expression of IL6 and IL8. The production of IL6 and IL8 inhibited ZIKV replication. Meanwhile, it possibly activated NF-κB signaling pathway further through a positive feedback regulation (40).

In summary, we propose a mechanism where ZIKV infection downregulates hsa_circ_0007321 expression, which typically acts as a sponge for miR-492. The subsequent increase in miR-492 then inhibits expression of NFKBID (IκBδ). Therefore, ZIKV replication reduces hsa_circ_0007321 to inhibit NFKBID expression, which in turn leads to the activation of the NF-κB signaling pathway and the production of proinflammatory cytokines that work to control ZIKV replication (Fig. 14). These results provide evidence that hsa_circ_0007321 and NF-κB may be potential therapeutic targets for inhibiting ZIKV infection, as well as other viruses.

## MATERIALS AND METHODS

### Cell culture and Zika virus infection

A549 (human non-small cell lung cancer cells), 293T (human embryonic kidney cells), and U251 (human glioma cells) were obtained from the West China Hospital of Sichuan University. The cells were grown in DMEM (Hyclone, USA) and supplemented with 10% fetal bovine serum (Biological Industries, Israel) and 1% mycoplasma prevention reagent (TransGen Biotech, China) at 37°C with 5% CO_2_ in an incubator. Zika virus (GZ01 strain) was kindly provided by Dr. Chengfeng Qin (Institute of Microbiology and Epidemiology, Beijing, China).

For ZIKV infection, cells were infected with ZIKV for 4 h at 37°C with 5% CO_2_ incubator, and then medium was aspirated. The cells were washed with phosphate-buffered saline (Hyclone, USA) and replaced with fresh culture medium.

### RNA-Seq and data analysis

The preparation of libraries and the procedure of RNA-Seq were performed by Novogene Co., Ltd (Beijing, China). To enrich pure circRNAs, linear RNAs were removed from the total RNAs using RNase R (Sigma, USA). Then, libraries were constructed according to the instructions of NEBNext Ultra RNA Library Prep Kit for Illumina (NEB, USA). The differentially expressed circRNAs with fold change >2 and *P* < 0.05 were identified using DESeq R package. All the sequence data are available through the GEO database with accession number GSE146423.

### RNA isolation, reverse transcription, and gene quantification

Total RNA was extracted from the cells using Trizol reagent (Invitrogen, USA). First-strand cDNA was synthesized using ReverTra Ace qPCR RT Master Mix (Toyobo, Japan) and was diluted with nuclease-free water. The qRT-PCR was carried out using SYBR Green Real-time Master Mix (Novoprotein, China) on a qTOWER^3^ Real-Time System (Jena, Germany). The mRNA level of each gene was calculated by using 2^−ΔΔCt^ method, normalized to GAPDH or U6. The sequences of the primers used in this study are listed in Table 1.

**TABLE 1:** Real-time PCR primer sequences

Gene name		Primer sequences (5′−3′)
ZIKV	Forward	GCAGAGCAACGGATGGGATA
	Reverse	ATGGTGGGAGCAAAACGGAA
IL-6	Forward	ACTCACCTCTTCAGAACGAATTG
	Reverse	CCATCTTTGGAAGGTTCAGGTTG
IL-8	Forward	CTTGGTTTCTCCTTTATTTCTA
	Reverse	GCACAAATATTTGATGCTTAA
NFKBID	Forward	GTGTACCGGCGTCTTGACATT
	Reverse	GTGAGGCCCTCGAAGTCTCT
GAPDH	Forward	GCCTCCTGCACCACCAACTG
	Reverse	ACGCCTGCTTCACCACCTTC
U6	Forward	CTCGCTTCGGCAGCACA
	Reverse	AACGCTTCACGAATTTGCGT

### Mimic, inhibitor, siRNA, and transfection

The small interfering RNA and negative control were designed and synthesized by GenePharma (Shanghai, China). MiR-492 mimics, miR-492 inhibitors, and their corresponding negative controls were purchased from RiBoBio (Guangzhou, China) and were transfected into cells using Lipofectamine RNAiMAX reagent (Thermo Fisher Scientific, USA) according to the manufacturer’s instructions.

### TNF-α and BAY 11-7082 treatment

The TNF-α (Sangon Biotech，China) was diluted to 100 ng/µL with nuclease-free water according to the instructions and was stored at −20°C. For TNF-α treatment, TNF-α was diluted to 25 ng/mL, and then cells were stimulated for 12 h for Western blots and dual-luciferase reporter gene assays. For the immunofluorescence assays, cells were stimulated for 1 h. The NF-κB inhibitor BAY 11-7082 (Beyotime, China) was diluted to 20 mM with dimethyl sulfoxide (Biofroxx, Germany) according to the instructions and was stored at −20°C. For BAY 11-7082 treatment, BAY 11-7082 was added to the cells to get the target final concentrations for 1 h. Then the cells were washed three times with PBS and replaced with fresh medium for further experimental treatments.

### ZIKV binding and entry assays

ZIKV binding and entry assays were performed as described before(41). For the binding assays, cells were infected at 4°C for 1 h, were washed with PBS to remove free viruses that did not bind to the cell surface, and were lysed accordingly. For the entry assays, cells were incubated at 37°C for 1 h.

### Western blotting and antibodies

Cells were lysed using radioimmune precipitation assay strong lysis buffer (Beyotime, China) containing PMSF (phenylmethanesulfonyl fluoride) protease inhibitor (Solarbio, China) and phosphatase inhibitor (TransGen Biotech, China). The lysates were centrifuged at 15,000 g for 15 min at 4°C, and the precipitates were removed. Then the protein concentrations were quantified by the BCA Protein Assay Kit (Beyotime, China). A total of 30 µg protein samples were boiled at 98°C for 5 min and loaded into SDS-PAGE gels, which were prepared using the PAGE Gel Rapid Preparation Kit (EpiZyme, China). Then the protein bands were transferred to PVDF membranes (Millipore, United States) and were blocked with 5% bovine serum albumin (BSA; Solarbio, China) for 2 h at room temperature. Targeted proteins were detected using specific primary antibodies as follows: anti-Phospho-NF-κB p65 and anti-NF-κB p65 (Cell Signaling Technology, USA), anti-IκBα (Affinity, China), anti-ZIKV NS1 (GeneTex, USA), anti-NFKBID (Abcam, UK), and anti-GAPDH (Proteintech, China). The membranes were washed three times with TBST buffer and were incubated with horseradish peroxidase (HRP)-conjugated goat anti-mouse IgG or anti-rabbit IgG secondary antibodies (Proteintech, China) at room temperature for 1 h. The membrane was incubated with chemiluminescence HRP substrate (Millipore, United States), and the protein bands were detected by using a ChemiDoc imaging system (Bio-Rad, United States). Densitometry analysis of Western blot results was performed with Image J software. GAPDH was used as a loading control, and protein expression was normalized to the GAPDH levels.

### CircRNA identification and subcellular localization

Total RNAs were extracted using Trizol reagent, and genomic DNA was isolated using TIANamp Genomic DNA Kit (TianGen, China) from A549 cells, respectively. RNA was reverse transcribed to cDNA and detected by qRT-PCR together with gDNA. The amplified products were verified by agarose gel electrophoresis and photographed by Uvsoloztouch (Jena, Germany). To verify the characteristic of circRNA resistance to RNase R, we treated total RNAs with RNase R at 37°C for 15 min according to the instructions (Invitrogen, USA). cDNA was synthesized, and the expression of hsa_circ_0007321 and the parental gene DIS3L2 mRNA were detected by qRT-PCR.

Nucleus-plasma separation assays were performed using Cytoplasmic and Nuclear RNA Purification Kit (Norgen Biotek, Canada) to determine the subcellular localization of hsa_circ_0007321 in A549 cells. Cells were washed with PBS and lysed by pre-cooled Lysis Buffer J for 5 min. Then the cytoplasmic and nuclear fractions were separated by centrifuging the lysates at 14,000 g for 10 min at 4°C. The supernatant containing cytoplasm was carefully transferred to a new tube, and the nuclear pellet was resuspended in buffer SK. Then, RNAs were isolated both from cytoplasmic and nuclear fractions. Expressions of GAPDH, U6, and hsa_circ_0007321 were detected by qRT-PCR. GAPDH was used as a cytoplasmic control gene, and U6 was used as a control gene in the nucleus.

### RNA pull-down assay

A549 cells were seeded in a 10-cm dish with 90% confluence and were washed with PBS containing RNase inhibitor (Solarbio, China) twice. Then cells were lysed and incubated with 3 µL of a 100-µM biotin-labeled hsa_circ_0007321 probe or a negative control probe (GenePharma, China) at 37°C for 3 h, respectively. Then the streptavidin-coated magnetic beads were added into the lysates and were incubated for 1 h. The magnetic beads were washed with wash buffer 1 and wash buffer 2 (GenePharma, China), and then the supernatant was removed. Lysis buffer and proteinase K (Beyotime, China) were added to the pellets and were incubated at 65°C for 1 h. Finally, the RNA bound on magnetic beads was extracted using Trizol reagent to determine the relative expression of miR-492 and miR-548c-3p. The sequences of the probes used in this study are listed in Table 2.

**TABLE 2:** The sequences of probes

Probe name	Probe sequences (5′−3′)
hsa_circ_0007321 probe	TGTTGTCATTCGCTTCCAATGCTCC
Negative control probe	CCTGCCTGTCTAACTCCGATTTTTG

### Dual-luciferase reporter assay

The database CircInteractome (https://circinteractome.nia.nih.gov/) was used to predict the possible binding sites between hsa_circ_0007321 and miR-492. The dual-luciferase reporter gene plasmids used in this study were purchased from GenePharma, China. The full-length hsa_circ_0007321 was constructed into a dual-luciferase reporter gene vector pmirGLO to obtain pmirGLO-circ_0007321 WT. The pmirGLO-circ_0007321-MUT plasmid was constructed based on the WT plasmid with a 5′-CAGGTCC-3′ to 5′-GTCCAGG-3′ mutation at the binding site. Meanwhile, the putative binding sites between NFKBID 3′ UTR and miR-492 seed sequence were predicted by Targetscan. Wild-type or mutant NFKBID 3′ UTR sequences containing the binding sites of miR-492 seed sequence were constructed into the reporter vector pmirGLO. The reporter plasmids were co-transfected into 293T cells with miR-492 mimic or the corresponding negative control using Lipofectamine 3000 transfection reagent (Invitrogen, USA). The luciferase activities were measured on an EnSpire Multimode Plate Reader (PerkinElmer, United States) at 48 h post-transfection. Binding between hsa_circ_0007321 and miR-492, miR-492 and the target gene NFKBID was analyzed by the firefly/*Renilla* luciferase activity ratio.

To assess the effect of miR-492 on NF-κB activation, we co-transfected the luciferase reporter plasmids containing NF-κB promoter and firefly luciferase gene (pNF-κB-Luc) (Huayueyang Biotech, China) together with miR-492 mimic or the negative control into 293T cells. pRL-TK (expression *Renilla* luciferase) was also transfected into the cells as an internal control. The luciferase activities were measured using the Dual-Luciferase Reporter Assay System (Promega, USA). Relative luciferase activity was calculated by dividing the firefly luciferase value by *Renilla* luciferase value.

### Immunofluorescence

The medium was removed, and the cells were fixed with 400 µL of 4% paraformaldehyde (Biosharp, China) for 30 min and were permeabilized with 0.2% TritonX-100 (Thermo Fisher Scientific, USA) for 15 min at room temperature. The cells were then blocked with 5% BSA at 37℃ for 1 h and incubated with NF-κB p65-specific primary (Cell Signaling Technology, USA) antibody or ZIKV capsid-specific primary antibody (GeneTex, USA) at 1:500 dilutions overnight at 4℃. After being washed with PBS, the cells were incubated with the secondary antibody (Proteintech, China) at 1:300 dilution at 37°C for 1 h. Cells were stained with DAPI (Solarbio, China) for 5 min before being observed by the fluorescence microscope. The images were taken under an inverted fluorescence microscope and were analyzed with Image J software.

### Cell viability assay

Cells were seeded in 96-well plates and treated according to the protocols for each experiment. After treatment, cell viability was detected by the Cell Counting Kit-8 assay (Biosharp, China) according to the manufacturer’s instructions. The absorbance at 450 nm was determined by an EnSpire Multimode Plate Reader (PerkinElmer, United States). The cell viability was calculated by using (OD_experiment_ − OD_blank_)/(OD_control_ − OD_blank_) × 100%.

### Statistical analysis

Statistical analyses and mapping were performed with GraphPad Prism 8 software. All experiments were repeated at least three times. Data were expressed as mean  ±  SD. Two-tailed Student’s *t*-tests were performed to determine the difference between two groups, and one-way ANOVA was used for comparison among more than three groups. *P*-value  <0.05 was considered as statistically significant (ns represents *P* > 0.05; * represents *P* < 0.05; ** represents *P* < 0.01; *** represents *P* < 0.001).
